# Comprehensive genome-wide analysis of calmodulin-binding transcription activator (CAMTA) in *Durio zibethinus* and identification of fruit ripening-associated *DzCAMTA*s

**DOI:** 10.1186/s12864-021-08022-1

**Published:** 2021-10-14

**Authors:** Zahra Iqbal, Mohammed Shariq Iqbal, Lalida Sangpong, Gholamreza Khaksar, Supaart Sirikantaramas, Teerapong Buaboocha

**Affiliations:** 1 Molecular Crop Research Unit, Department of Biochemistry, Chulalongkorn University, Bangkok, Thailand; 2 Amity Institute of Biotechnology, Amity University, Lucknow Campus, Lucknow, Uttar Pradesh India; 3 Omics Sciences and Bioinformatics Center, Faculty of Science, Chulalongkorn University, Bangkok, Thailand

**Keywords:** Auxin, CAMTA, Durian, Ethylene, Fruit ripening, Transcriptional regulation

## Abstract

**Background:**

Fruit ripening is an intricate developmental process driven by a highly coordinated action of complex hormonal networks. Ethylene is considered as the main phytohormone that regulates the ripening of climacteric fruits. Concomitantly, several ethylene-responsive transcription factors (TFs) are pivotal components of the regulatory network underlying fruit ripening. Calmodulin-binding transcription activator (CAMTA) is one such ethylene-induced TF implicated in various stress and plant developmental processes.

**Results:**

Our comprehensive analysis of the CAMTA gene family in *Durio zibethinus* (durian, Dz) identified 10 CAMTAs with conserved domains. Phylogenetic analysis of DzCAMTAs, positioned DzCAMTA3 with its tomato ortholog that has already been validated for its role in the fruit ripening process through ethylene-mediated signaling. Furthermore, the transcriptome-wide analysis revealed *DzCAMTA3* and *DzCAMTA8* as the highest expressing durian *CAMTA* genes. These two *DzCAMTAs* possessed a distinct ripening-associated expression pattern during post-harvest ripening in Monthong, a durian cultivar native to Thailand. The expression profiling of *DzCAMTA3* and *DzCAMTA8* under natural ripening conditions and ethylene-induced/delayed ripening conditions substantiated their roles as ethylene-induced transcriptional activators of ripening. Similarly, auxin-suppressed expression of *DzCAMTA3* and *DzCAMTA8* confirmed their responsiveness to exogenous auxin treatment in a time-dependent manner. Accordingly, we propose that *DzCAMTA3* and *DzCAMTA8* synergistically crosstalk with ethylene during durian fruit ripening. In contrast, *DzCAMTA3* and *DzCAMTA8* antagonistically with auxin could affect the post-harvest ripening process in durian. Furthermore, *DzCAMTA3* and *DzCAMTA8* interacting genes contain significant CAMTA recognition motifs and regulated several pivotal fruit-ripening-associated pathways.

**Conclusion:**

Taken together, the present study contributes to an in-depth understanding of the structure and probable function of *CAMTA* genes in the post-harvest ripening of durian.

**Supplementary Information:**

The online version contains supplementary material available at 10.1186/s12864-021-08022-1.

## Background

Fruit ripening is a highly coordinated, complex, and programmed process that involves various physiological modifications, such as changes in color, texture, aroma, quality, and nutritional value [1]. Depending upon the mechanism of action during ripening, fruits are classified into climacteric and non-climacteric groups. Autolytic increase in ethylene biosynthesis triggers the ripening of climacteric fruits, such as tomatoes and apples. In concurrence, several ethylene biosynthesis and signaling genes had been reported to play crucial roles in fruit ripening [2–4]. In contrast, the ripening of non-climacteric fruits such as grapes and strawberries is an ethylene-independent process [5, 6]. The process of fruit ripening is tightly regulated by transcription factors (TFs) [7, 8]. Fruit ripening is also affected by environmental conditions, such as biotic and abiotic stresses [9]. Additionally, calcium (Ca^2+^) has been implicated in maintaining fruit firmness and delaying ripening [10, 11]. Often, molecular mechanisms mediating plant responses to environmental and hormonal signals involve signaling cascades in which Ca^2+^ acts as a pivotal second messenger [12, 13]. Perturbations in cytosolic Ca^2+^ levels are sensed and interpreted by an array of Ca^2+^-binding proteins that function as signal sensors [14, 15]. Calmodulin (CaM) is a well-characterized Ca^2+^ sensor, which is known to modulate target proteins such as TFs [16], including the well-known CaM-binding transcription activators (CAMTAs) [17, 18]. CAMTAs, also known as signal responsive (SR) proteins [18] or ethylene-induced CaM-binding proteins (EICBP) [17] have been extensively characterized as the largest CaM-binding TF family [18]. The CAMTA proteins are reported to possess six functional domains [19], namely (i) CG1 domain, unique DNA binding sequence; (ii) NLS, nuclear localization signal to target the protein into the nucleus, (iii) TIG domain, implicated in non-specific DNA interactions, (iv) CaMBD, CaM-binding domain for the interaction of CaM with CAMTA; (v) ankyrin (ANK) repeat, contributing to protein-protein interactions; and (vi) IQ motif, region of low complexity for binding CaM and CaM-like proteins. CAMTAs were first reported as an early ethylene-responsive (*NtER*) gene implicated in ethylene-regulated plant death and senescence, indicating its probable role in prolonging the shelf life of crops [18]. Later, a homolog of *NtER1* and five related genes in *Arabidopsis* (*AtSR*s) were shown to play significant roles in several signal transduction pathways, including hormonal, developmental, and environmental cues [20]. Since then, CAMTAs have been reported to play significant roles in various biotic stresses, abiotic stresses, and plant development [21–24]. CAMTAs are present in a number of eukaryotic genomes, including *Arabidopsis thaliana* [25], *Oryza sativa* [26], *Solanum lycopersicum* [27], *Vitis vinifera* [28], *Medicago truncatula* [29], *Fragaria ananassa* [30], *Glycine max* [31], *Zea mays* [32], *Brassica napus* [33], *Populus trichocarpa* [34], *Gossypium hirsutum* [24], *Nicotiana tabacum* [35], *Citrus sinensis* [36], *Musa acuminata* [37], *Phaseolus vulgaris* [38], and *Linum usitatissimum* [39]*.* The underpinning mechanisms of CAMTAs in drought, cold, and salt tolerance has been reasonably elucidated [23, 40–42]. Similarly, *Arabidopsis thaliana CAMTA3* has been comprehensively studied for its involvement in biotic stress responses [22, 43, 44]. Additionally, the phytohormonal regulation of CAMTAs is extended to auxins [32, 45, 46], brassionosteroids [47], ethylene [27, 30, 48], abscisic acid (ABA) [32, 41], methyl jasmonate [32], and salicylic acid [32, 40, 49]. Apart from the notable involvement of CAMTAs in stress and hormonal biology, a unique involvement of *Solanum lycopersicum* CAMTAs (*SlSR*s) in fruit development and ripening has been reported [27]. All seven *SlSR*s were highly and differentially expressed during fruit development and ripening. The expression profile of *SlSR*s significantly varied in *rin* mutant (ripening mutant) in comparison to the wildtype. Contradictorily, the alterations in expression of *SlSR*s were minor for the other two ripening mutants (*nor* mutant and *Nr* mutant*)*. In addition, the treatment of mature green wildtype fruit with ethylene transiently enhanced the expression of all *SlSR*s [27].

To investigate the role of Ca^2+^-regulated CAMTAs in fruit ripening, we selected durian, a tropical fruit crop native to Southeast Asia. Durian is an economically important fruit that has recently gained huge popularity in international markets due to its unique odor, formidable spiny husk, and distinct flavor. It is considered an imperative export perishable item in Southeast Asia. Thailand is the top exporter of durian in Southeast Asia and other international markets. More than 200 durian cultivars are known; however, only a few of them are commercially cultivated and exported. In Thailand, four durian cultivars, Monthong (*D. zibethinus* Murr. ‘Monthong’), Chanee (*D. zibethinus* Murr. ‘Chanee’), Phuangmanee (*D. zibethinus* Murr. ‘Phuangmanee’), and Kanyao (*D. zibethinus* Murr. ‘Kanyao’) are of great popularity in the local and international markets [50]. Monthong is a slow-ripening cultivar with less sweet taste, mild odor, creamy texture, and light-yellow pulp. It requires approximately five days after harvest (mature stage) to ripen. Once harvested, durian has a very limited shelf life. Hence, the ripening processes associated with durian is of great agronomic and economic significance for the agricultural industry. In Thailand, durian harvesting is performed at the mature stage (approximately 105 days for Monthong). Maintaining the flavor and nutritional value of durian fruit along with a longer shelf life poses a challenge for the industry. In neoteric times, research encircling the extended shelf life of fruits has gained momentum [51]. However, the underpinning mechanistic details fundamental to the fruit ripening process remain obscure. In addition, despite the fact that durian is gaining importance as an economic crop, genomic and molecular research on this crop is limited, especially with regard to the CAMTA TFs.

Coherently, the present study aimed to deepen our understanding of the structure and function of the CAMTA gene family in durian. This study identified ten DzCAMTAs in the durian genome. Comprehensive expression profiling of *DzCAMTAs* during durian post-harvest ripening revealed significant differential expression of *DzCAMTAs* and proposed the putative ripening-associated members. Our results evidently positioned *DzCAMTA3* and *DzCAMTA8* as the key members of the regulatory framework underlying the post-harvest ripening of durian. To our knowledge, the present study is the pioneer report examining the CAMTA gene family and its roles in the post-harvest ripening of this climacteric fruit.

## Results

### Genome-wide identification of durian *CAMTA* genes, their domain organization, gene structure, and motif composition

**Fig. 1:**
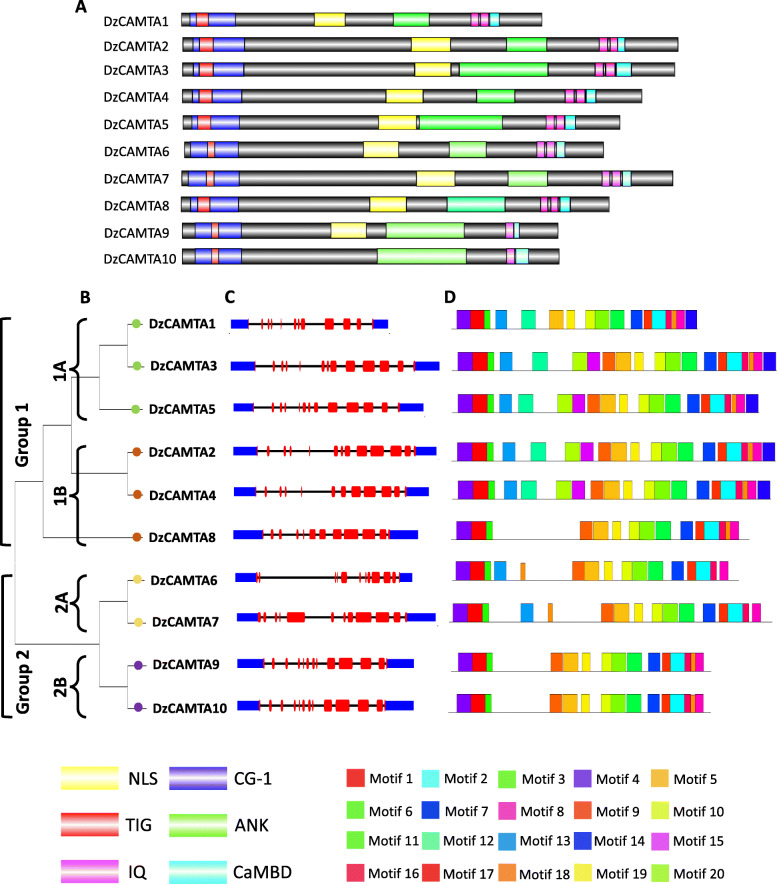
Domain, gene and motif organization for 10 DzCAMTAs. **(A)** Schematic representation of functional domains of 10 putative DzCAMTAs. Bioinformatics analysis of functional conserved domains was performed by CD search, and searches in Pfam, and InterPro databases. CaMBD was searched in Calmodulin Target Database. NLS was specifically searched in NLS mapper and Motif Scan databases. The domain structure for 10 putative DzCAMTAs was drawn using Illustrator for Biological Sequences software. **(B)** Phylogenetic tree of 10 putative DzCAMTAs constructed with ML method using 1000 bootstrap value. **(C)** Schematic representation of the exon and intron organization of 10 putative *DzCAMTA* genes. The red boxes represent the exons, the black lines represent the introns, and the blue boxes represent the upstream and downstream regions. **(D)** The conserved protein motifs in the 10 putative DzCAMTAs. Each motif is represented with a specific color

HMMER search was performed with the conserved DNA-binding CAMTA domain to identify CAMTA orthologs in durian species (Additional file 1-Table S1). After removal of redundant and partial sequences, 10 CAMTAs were identified in the durian genome (Table 1). Subsequently, all the putative *CAMTA* genes in durian were confirmed through similarity and conserved domain searches against the Pfam, NCBI conserved domain, and InterPro databases (Table 1 and Additional file 1-Table S2). The length of the identified durian CAMTA proteins varied from 891 amino acids to 1083 amino acids. The theoretical pI varied from 5.39 to 8.1, whereas the molecular weight ranged from 99,995.47 Da to 122,683.04 Da. The number of exons in *DzCAMTA*s was quite similar (12–13 per gene), possibly indicative of functional redundancy. Additionally, all the identified *DzCAMTA*s were presumably localized to the nucleus with a high degree of reliability (Table 1). This implies that the physicochemical characteristics of *DzCAMTA*s were highly conserved, with few obvious exceptions. For the standard annotation of the ten *DzCAMTA*s, we followed the numeric nomenclature system applied to *AtCAMTA*s. The MSA of DzCAMTAs by ClustalX and their sequence scanning using the Pfam, NCBI conserved domain, and InterPro databases revealed that all the DzCAMTAs were composed of the reported CAMTA domains (Fig. 1A and Additional file 2). All DzCAMTAs contained the signature CG-1 domain, IQ motif, CaMBD, and ankyrin repeat (Fig. 1A). The ten identified DzCAMTAs also contained a bipartite nuclear localization signal (NLS) at the N-terminus (within the CG-1 domain), suggesting that this domain might contribute to a signal that directs the import of DzCAMTAs to the nucleus. Moreover, with the exception of DzCAMTA10, the remaining DzCAMTAs comprised the TIG domain. It has been previously reported that *Arabidopsis thaliana, Arabidopsis lyrata, Gossypium raimondii, Gossypium hirsutum, Gossypium arboretum,* and *Capsella rubella* also encode non-TIG CAMTAs [24, 52]. The TIG domain originated first in the ancestors of the land plants. Mutation of some vital amino acids in the TIG domain rather than complete deletion of the TIG domain occurred in the non-TIG CAMTAs during the course of evolution [52]. Thus, non-TIG CAMTAs might contribute to the expansion of DzCAMTAs. The number of IQ motif in DzCAMTAs varied from 1 to 2 and were consistently found at the C-terminus of the protein (Fig. 1A). Nonetheless, the present study in durian validated the reported functional domains of CAMTA TF [19]. The evolutionary relationship among DzCAMTAs was deduced by constructing an ML phylogenetic tree with stringent bootstrap values (Fig. 1B). The DzCAMTAs were divided into two groups. Group 1 was further divided into two sub-groups (sub-group 1A and sub-group 1B). Similarly, group 2 was also divided into two sub-groups (sub-group 2A and sub-group 2B). In sub-group 1A, *DzCAMTA3* and *DzCAMTA5* shared a similar exon-intron organization (13 exons and 12 introns each); whereas in sub-group 1B, *DzCAMTA2* and *DzCAMTA4* shared a related exon-intron organization (12 and 13 exons respectively) (Fig. 1C). Intriguingly, sub-group 2B *DzCAMTA*s (i.e., *DzCAMTA9* and *DzCAMTA10*) shared a strikingly high similarity in terms of their exon-intron organization and distribution pattern (13 exons and 12 introns of almost similar lengths) (Fig. 1C). The similar exon-intron organization of these *DzCAMTA*s might be an indication of their functional redundant behavior or their origin as paralogous genes with different functions. Sub-group 2A *DzCAMTA6* possessed the largest introns. Whether this particular gene exhibits a distinct function from the rest of the *DzCAMTA*s remains a subject for further study. Further, MEME tool was used to comprehend the motif conserveness among all 10 *Dz*CAMTAs. Twenty putative conserved motifs were identified by MEME in DzCAMTAs (Fig. 1D and Additional file 3). Motifs 1, 4, and 11 that hit the Pfam, NCBI conserved domain, and InterPro databases were identified as CG-1 domain (Additional file 1- Table S3). Motifs 2 and 3 were the IQ motif and ankyrin repeat-containing domain, respectively (Additional file 1-Table S3). Motifs 5 and 9 were associated with the TIG domain (Additional file 1- Table S3). Motifs 1, 4, and 11 (the red, purple, and green motifs; CG-1 or the signature CAMTA motif) were identified in all the DzCAMTAs and represented the conserved CAMTA domain.

**Table 1:** The CAMTA genes in *Durio zibethinus* and properties of the deduced proteins

Gene name	Corresponding gene ID	Corresponding protein ID	Locus ID	Scaffold	strand	ORF length (bp)	Protein length (aa)	Molecular Weight (dalton)	***p***I value	No. of Exon	Subcellular localization
DzCAMTA1	XM_022870778.1	XP_022726513.1	LOC111282620	NW_019168048.1	+	2676	891	99,995.47	6.74	12	Nucleus
DzCAMTA2	XM_022884479.1	XP_022740214.1	LOC111292222	NW_019168470.1	+	3252	1083	122,683.04	6.06	13	Nucleus
DzCAMTA3	XM_022888585.1	XP_022744320.1	LOC111295185	NW_019167849.1	+	3249	1082	121,828.47	5.8	13	Nucleus
DzCAMTA4	XM_022895339.1	XP_022751074.1	LOC111299855	NW_019167860.1	+	3153	1050	119,054.19	5.71	12	Nucleus
DzCAMTA5	XM_022907953.1	XP_022763688.1	LOC111309099	NW_019167915.1	–	3111	1036	116,782.75	5.64	13	Nucleus
DzCAMTA6	XM_022869391.1	XP_022725126.1	LOC111281753	NW_019168048.1	+	2958	985	109,475.52	5.56	12	Nucleus
DzCAMTA7	XM_022920611.1	XP_022776346.1	LOC111318006	NW_019167971.1	–	3252	1083	120,256.18	5.39	12	Nucleus
DzCAMTA8	XM_022905033.1	XP_022760768.1	LOC111306973	NW_019167904.1	–	3006	1001	111,703.91	7.21	12	Nucleus
DzCAMTA9	XM_022866124.1	XP_022721859.1	LOC111279200	NW_019168026.1	–	2745	914	103,362.48	7.85	13	Nucleus
DzCAMTA10	XM_022904915.1	XP_022760650.1	LOC111306911	NW_019167904.1	+	2745	914	102,912.3	8.1	13	Nucleus

### Phylogenetic relationship of durian CAMTAs with other plant species

**Fig. 2:**
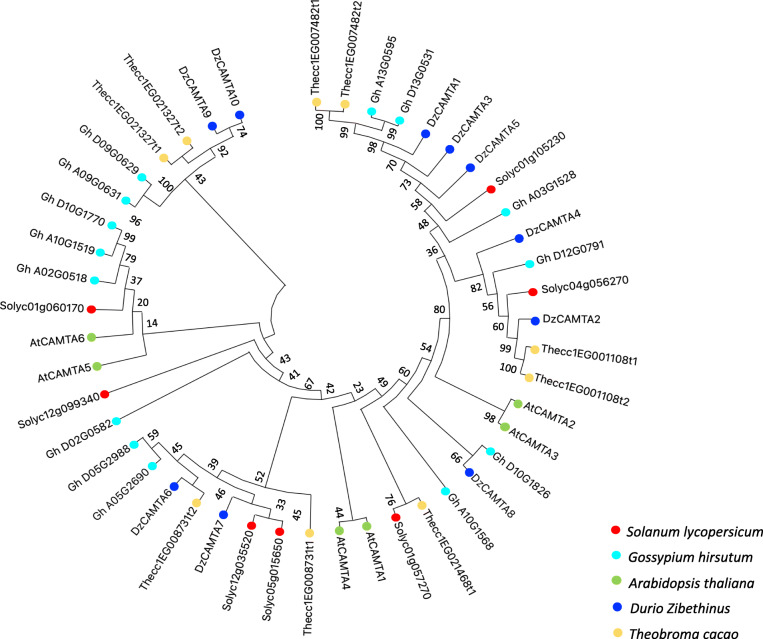
Phylogenetic tree of CAMTA gene family in *Durio Zibethinus* and four other representative plant species. The full-length protein sequences of *Arabidopsis thaliana, Solanum lycopersicum, Theobroma cacao, Gossypium hirsutum,* and *Durio Zibethinus* were aligned using MEGA X software. The phylogenetic ML tree was constructed with the JTT model and pairwise gap deletion using bootstrap test (bootstrap value = 1000) with default parameters. The numbers against the subsequent branches indicate the bootstrap values that support the adjacent nodes. Different species are represented by colored dots; red: *Solanum lycopersicum*, aqua: *Gossypium hirsutum*, green: *Arabidopsis thaliana*, dark blue: *Durio Zibethinus*, and yellow: *Theobroma cacao*

A phylogenetic tree was constructed to gain insights into the evolutionary relationship of DzCAMTAs with CAMTAs from other plant species. The evolutionary relationships among the 10 identified DzCAMTAs and CAMTAs from four different plant species (Additional file 1- Table S4) were analyzed using the bootstrap consensus ML tree. We performed MSA of 10 identified DzCAMTAs with 36 CAMTA protein sequences from four different plant species (families: Brassicaceae, Solanaceae, and Malvaceae). Subsequently, the DzCAMTAs distinctly clustered with all the other four plant species with strong bootstrap support (Fig. 2). One interesting observation was the clustering of DzCAMTA1, DzCAMTA3, and DzCAMTA5 with *Solanum lycopersicum* Solyc01g105230 (*SlSR1L*) that is well implicated in fruit ripening [27]. The DzCAMTAs and cotton CAMTAs (both plants belonging to the family Malvaceae) clustered together with very high reliability. This is indicative of the fact that DzCAMTAs and cotton CAMTAs originated from the same common ancestor. Consequently, it will be interesting to examine whether the CAMTA members of durian and Gossypium which clustered together function similarly, considering the involvement of Gossypium CAMTAs in fiber development [24].

### Scaffold distribution and duplication of durian *CAMTA*s

**Fig. 3:**
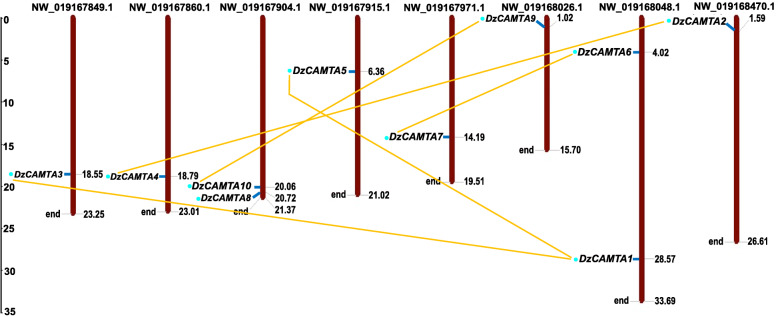
Scaffold position and paralogous durian *CAMTA* gene pairs. Physical map showing the position of *CAMTA* genes on durian scaffolds separately. The paralogous *DzCAMTA* genes are connected with yellow line. Horizontal grey line represents the position of each *DzCAMTA*. The scale is in mega bases (Mb)

Next, we assessed the physical locations of the *DzCAMTA* genes in the durian genome. Since, only the draft genome is available for durian (organized up to scaffold level), the scaffold positions of 10 *DzCAMTAs* were identified by BLASTN searches. The physical locations of each *DzCAMTA* gene are listed in Table 1. *DzCAMTA1* and *DzCAMTA6* (NW_019168048.1) as well as *DzCAMTA8* and *DzCAMTA10* (NW_019167904.1) were localized to the same scaffold (Fig. 3). The remaining six *DzCAMTAs* were distinctly localized to separate scaffolds. We further assessed the gene duplication events responsible for the expansion of durian CAMTAs. Based on high protein sequence identity and similarity, five pairs of putative paralogous *CAMTA* genes were identified in the durian genome (Fig. 3). Intriguingly, the paralogous durian *CAMTA* gene pairs clustered together in the phylogenetic tree, depicting a high degree of protein sequence identity (> 70%) (Fig. 2). It is worth mentioning that all five paralogous durian *CAMTA* gene pairs were located on different scaffolds. Moreover, the functional divergence and selection pressure of durian *CAMTA* genes were evaluated by Ka (nonsynonymous substitution), Ks (synonymous substitution), and Ka/Ks (nonsynonymous by synonymous substitution) ratios between the paralogs. The Ka/Ks ratios for paralogous durian *CAMTA* gene pairs were without exception < 1 (Additional file 1- Table S5). Hence, the duplicated durian genes had underwent strong purifying selection pressure accounting for the fact that they had not diverged much during the course of evolution. The duplication events for paralogous durian CAMTA gene pairs mainly occurred between 14.64 and 17.95 MYA (Additional file 1- Table S5).

### Durian *CAMTA*s encode CaM binding proteins

**Fig. 4:**
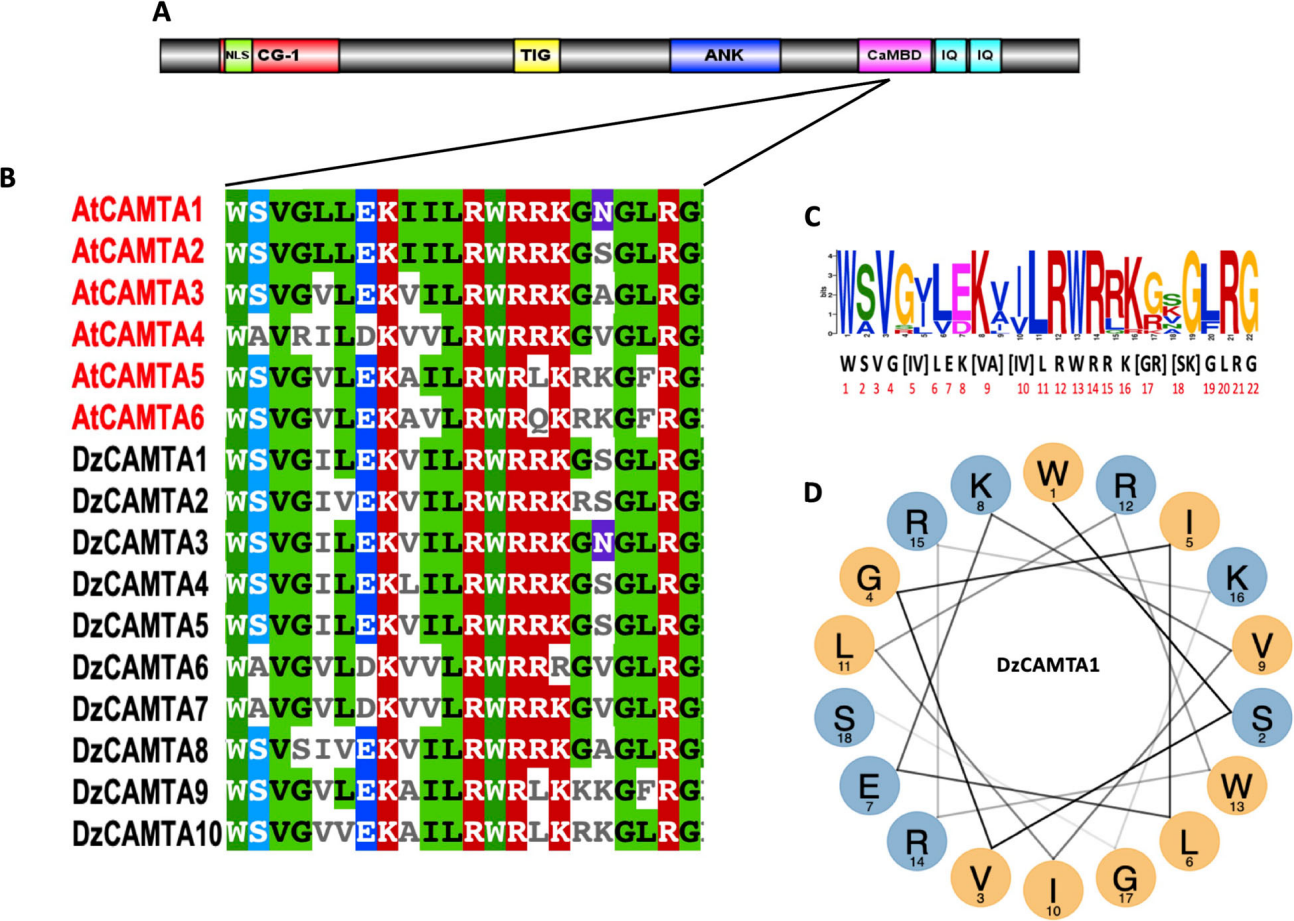
Conservation of calmodulin-binding domain (CaMBD) in 10 putative DzCAMTAs. **(A)** General schematic representation of domain organization of CAMTA protein. **(B)** Alignment of the putative conserved CaMBD of *Durio Zibethinus* with CaMBD of *Arabidopsis thaliana*. **(C)** Logo for the sequence of CaMBD in 10 putative DzCAMTAs with 6 *Arabidopsis thaliana* CAMTAs. **(D)** Helical wheel projection of the representative amphipathic α-helix structure in the predicted CaMBD of *Durio Zibethinus* CAMTA1*.* The non-polar amino acids (G, L, W, I, and V) are shown by yellow circles. The polar amino acids (S, E, R, and K) are shown by blue circles. W, L, G, I, and V represents the hydrophobic face

The unique feature of the CAMTA TFs is the presence of the CaMBD (Fig. 4A). The existence of CaMBD has been reported in CAMTAs from all organisms except *C. elegans* [25]. Previously, it has been shown in *Arabidopsis thaliana* [25], *Solanum lycopersicum* [27]*, Fragaria ananassa* [30]*,* and *Gossypium hirsutum* [24], that CaMBD is composed of a functional motif (WXVX(2)LXKX(2)[LF]RWRX[KR]X(3)[FL]RX). This motif is considered indispensable for CaM binding [25, 27]. To determine the conservation of CaMBD, the CaM-binding regions of DzCAMTAs were aligned with *Arabidopsis thaliana* CAMTAs. We identified the conserved sequence of CaMBD as WSVG[IV]LEK[VA][IV]LRWRRK[GR] [SK]GLRG (Fig. 4B and C), which was consistent with previous reports [24, 25, 27, 30]. Thus, the amino acid composition of CaMBD in *Arabidopsis thaliana* CAMTAs shares a very high sequence homology with its durian counterparts. For example, DzCAMTA6 and DzCAMTA7 have almost the same amino acid composition as AtCAMTA4, DzCAMTA3 has almost the same amino acid composition as AtCAMTA1, and DzCAMTA2 has almost the same amino acid composition as AtCAMTA2 (Fig. 4B). Additionally, the CaM-binding motif is capable of forming an amphipathic α-helix structure. We determined the amphipathic α-helical properties of the 10 identified DzCAMTAs. All 10 DzCAMTAs formed an amphipathic α-helix structure (for representation only DzCAMTA1 is shown). The results revealed an 18 amino acid long stretch to have a CaM-binding site (W, L, G, I, and V as the hydrophobic face) (Fig. 4D).

### Identification of *cis*-regulatory elements in the promoter of DzCAMTAs

**Fig. 5:**
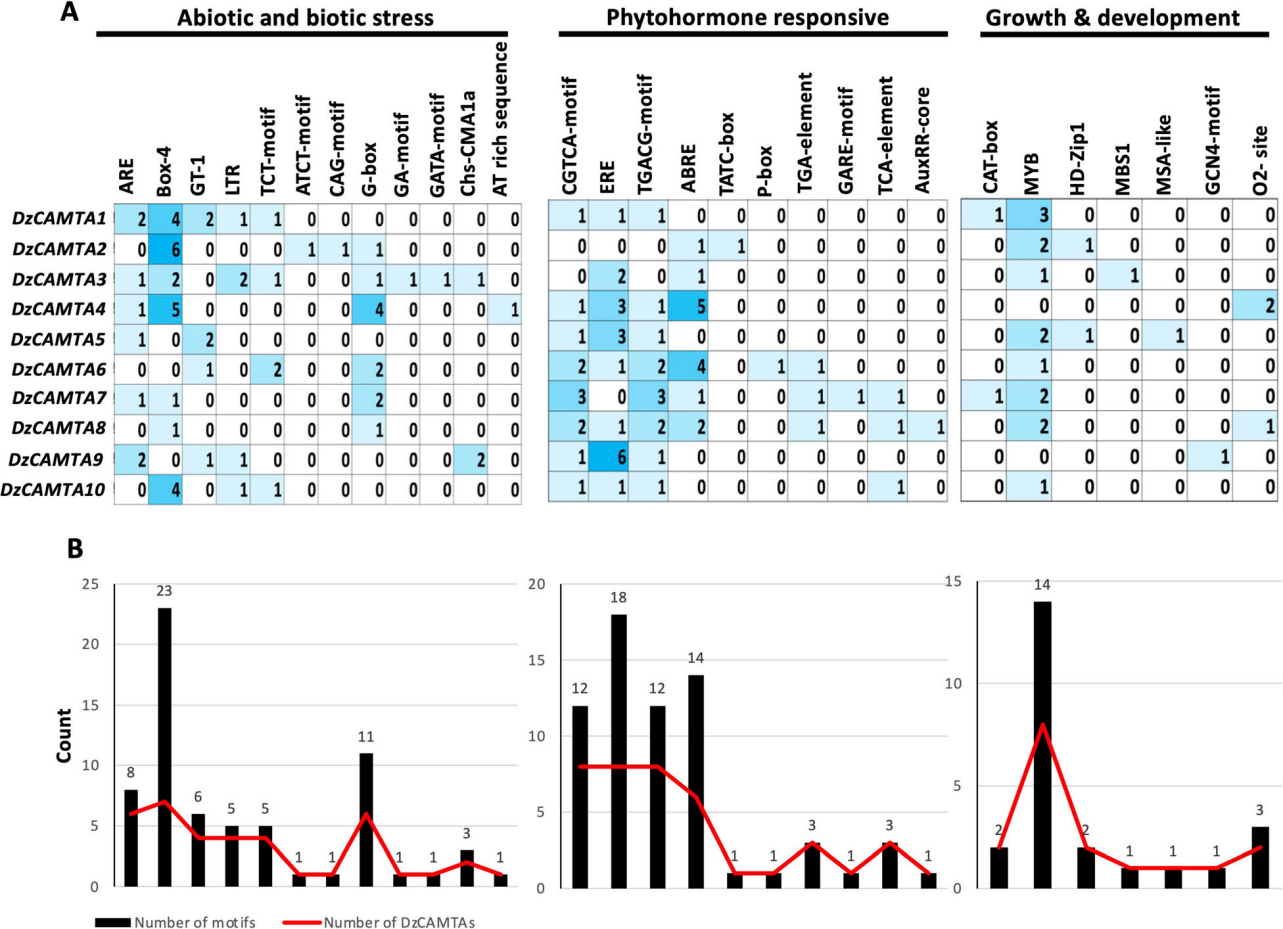
*Cis-*acting element analysis of the promoter regions of 10 putative *DzCAMTA* genes. **(A)** Counts of *cis-*acting elements in 1000 bp upstream region to the TSS in 10 *DzCAMTA* genes. **(B)** Total number of *DzCAMTA* genes (red line) with the corresponding number of *cis-*acting elements (black bar). The *cis-*acting elements were classified into three major groups depending upon their functional annotation namely; abiotic and biotic stress, phytohormone responsive, and growth and development

The identification of *cis*-regulatory elements in the promoter regions of the genes of interest provides important information regarding the gene function and regulation. The promoter sequences (1000 bp upstream to the transcription start site) of 10 *DzCAMTA*s were analyzed using the PlantCARE database [53] and the PLACE database [54] for the identification of *cis*-regulatory elements. *Cis*-acting elements implicated in abiotic and biotic stress, phytohormone regulation, and plant growth and development were identified in the promoters of the 10 *DzCAMTAs* (Fig. 5A and B, Additional file 1- Table S6). The GT-1 motif was found in four *DzCAMTAs (DzCAMTA1*, *DzCAMTA5*, *DzCAMTA6,* and *DzCAMTA9*), which has been extensively linked to salt and osmotic stresses in *Arabidopsis thaliana* [55]. Similarly, G-box, which is well linked with bHLHs and bZIPs [56] TFs, was also found to be enriched in the promoters of six *DzCAMTA* genes *(DzCAMTA2*, *DzCAMTA3*, *DzCAMTA4*, *DzCAMTA6*, *DzCAMTA7*, and *DzCAMTA8*). It has been previously reported that the G-box is involved in the regulation of plant stress responses and early leaf senescence [56, 57]. Furthermore, it was interesting to note the presence of a few light-responsive *cis-*elements (LREs) in almost all the *DzCAMTAs.* These included GATA-box (*DzCAMTA3*), G-box *(DzCAMTA2*, *DzCAMTA3*, *DzCAMTA4*, *DzCAMTA6*, *DzCAMTA7*, and *DzCAMTA8*), TCT motifs *(DzCAMTA1*, *DzCAMTA3*, *DzCAMTA6*, and *DzCAMTA10*), Box-4 (*DzCAMTA1*, *DzCAMTA2*, *DzCAMTA3*, *DzCAMTA4*, *DzCAMTA7*, *DzCAMTA8*, and *DzCAMTA10*), and chs-CMA1a *(DzCAMTA3* and *DzCAMTA9*) (Fig. 5A and B). This is probably indicative of the intersecting impact of light on the fruit ripening process during post-harvest handling [58, 59]. However, promoter sequencing can provide a better and more reliable information regarding the binding sites. Phytohormonal *cis-*acting elements implicated in the regulation of MeJA-responsive element - CGTCA motif and TGACG motif [60], SA-responsive element - TCA element [61], and ABA-responsive element - ABRE [62] were found to be enriched in the promoters of 8, 3, and 6 *DzCAMTA* genes, respectively. The gibberellin-responsive element- GARE motif, TATC box, and P-box [63] were present each in only one *DzCAMTA* gene. Similarly, the auxin-responsive element- AuxRR-core and TGA element [64] were observed in 1 and 3 *DzCAMTA* genes, respectively. As expected, the ethylene-responsive element (ERE) [65] was highly enriched in a high number of *DzCAMTA* genes (*DzCAMTA1*, *DzCAMTA3*, *DzCAMTA4*, *DzCAMTA5*, *DzCAMTA6*, *DzCAMTA8*, *DzCAMTA9*, and *DzCAMTA10*) (Fig. 5A and B). This indicates the probable cross-wired mesh of ethylene signaling in fruit ripening processes [4] through the involvement of *DzCAMTA*s. Considering the *cis-*acting elements in plant growth and development, MYB binding sites were found in almost all the *DzCAMTAs* (*DzCAMTA1*, *DzCAMTA2*, *DzCAMTA3*, *DzCAMTA5*, *DzCAMTA6*, *DzCAMTA7*, *DzCAMTA8*, and *DzCAMTA10*) while the GCN4_motif involved in endosperm expression [63] was enriched in *DzCAMTA9*. The leaf development-related *cis*-acting elements HD-Zip1 [66] and the zein metabolism regulation element (O2 site) were present in *DzCAMTA2* and *DzCAMTA5*, and in *DzCAMTA4* and *DzCAMTA8*, respectively*.* Additionally, CAT-box (meristem expression-related) was present in *DzCAMTA1* and *DzCAMTA7,* and MSA-like motifs (cell cycle regulation-related) was present in *DzCAMTA5* (Fig. 5A and B). Perhaps, the presence of these motifs is just indicative of the probable molecular regulation which can be further affirmed through sequencing.

### Differential expression of *DzCAMTA*s during post-harvest ripening of durian ‘Monthong’

**Fig. 6:**
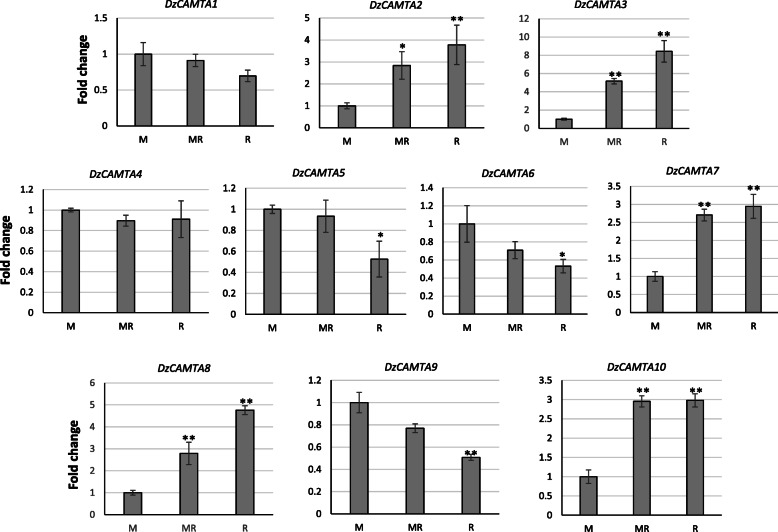
Real-time RT-PCR validation of 10 putative *DzCAMTA* genes in *Durio Zibethinus* pulp at three different stages (Mature-M, Midripe-MR, and Ripe-R) during post-harvest ripening. Data are the mean ± SE of three different biological replicates and three technical replicates. Asterisk indicates significant difference from values of M stage at *P* < 0.05 (*) *P* < 0.01(**) by t-test

We analyzed the expression pattern of the 10 *DzCAMTA*s at three stages of post-harvest ripening, namely mature, mid-ripe, and ripe by qRT-PCR (Fig. 6). Notably, 8 out of 10 *DzCAMTA*s were significantly expressed in fruit pulp and exhibited a ripening-associated pattern. These eight *DzCAMTA*s (*DzCAMTA2*, *DzCAMTA3*, *DzCAMTA5*, *DzCAMTA6*, *DzCAMTA7, DzCAMTA8*, *DzCAMTA9*, and *DzCAMTA10*) were considered as putative ripening-associated TFs. *DzCAMTA2*, *DzCAMTA3*, *DzCAMTA7*, *DzCAMTA8*, and *DzCAMTA10* were up-regulated during post-harvest ripening. In contrast, the transcripts for *DzCAMTA5*, *DzCAMTA6*, and *DzCAMTA9* were down-regulated over the course of post-harvest ripening. *DzCAMTA1* and *DzCAMTA4* did not show any significant changes in the expression levels. Since the expression levels of *DzCAMTA1* and *DzCAMTA4* did not significantly vary during the stages of post-harvest ripening, these two *DzCAMTA*s were not considered as ripening-associated TFs. With the exception of *DzCAMTA2* and *DzCAMTA4*, the expression of all *DzCAMTA* genes correlated with the transcriptome data. The transcript abundance of all *DzCAMTA*s was also observed at the pre-harvest stage (Immature1-IM1 and Immature 2-IM2) (Additional file 4). Except for *DzCAMTA1* and *DzCAMTA7*, no other *DzCAMTA* was induced significantly prior to fruit harvesting. Interestingly, in comparison to other *DzCAMTA*s, *DzCAMTA3* and *DzCAMTA8* were highly expressed (in comparison to the other six ripening-associated *DzCAMTA*s) at the ripe stage. The expression levels of *DzCAMTA3* and *DzCAMTA8* were also correlated with the transcriptome data (Additional file 5- Fig. A, B, and C). Thus, *DzCAMTA3* and *DzCAMTA8* were selected for further analysis. The transcript abundance of *DzCAMTA3* and *DzCAMTA8* (the two highly expressed putative ripening-associated *DzCAMTA*s) was further investigated under ethylene and auxin treatments.

### Validation of ripening-associated *DzCAMTA*s under three ripening treatments and auxin treatment

**Fig. 7:**
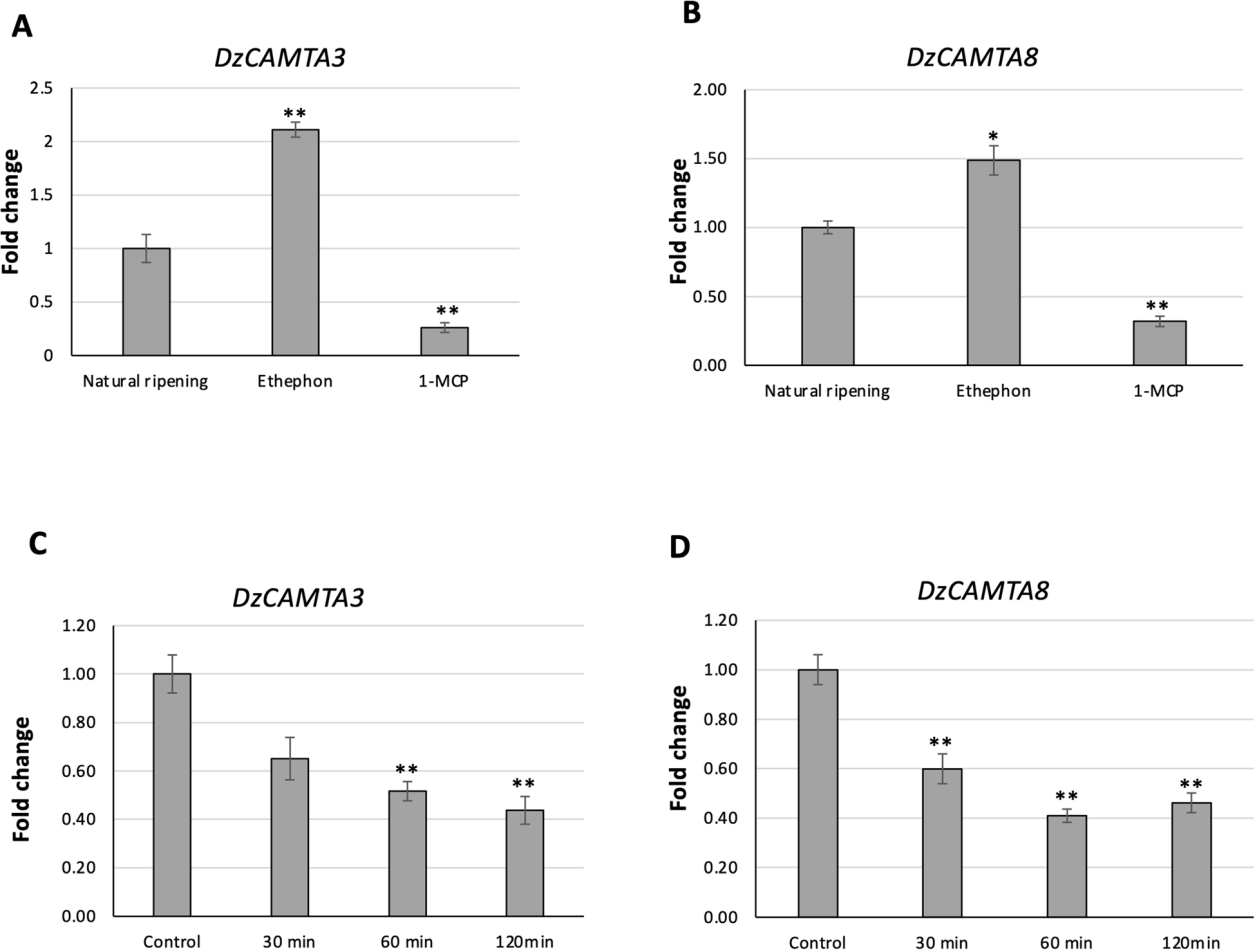
Ethylene and auxin responsiveness of *DzCAMTA3* and *DzCAMTA8*. **(A)** *DzCAMTA3* expression under three different ripening treatments: natural (control), ethylene-induced, and 1-MCP-delayed ripening. **(B)** *DzCAMTA8* expression under three different ripening treatments: natural (control), ethylene-induced, and 1-MCP-delayed ripening. **(C)** *DzCAMTA3* expression at 0 min, 30 min, 60 min, and 120 min of IAA treatment. **(D)** *DzCAMTA8* expression at 0 min, 30 min, 60 min, and 120 min of IAA treatment. Data are the mean ± SE of three different biological replicates and three technical replicates. Asterisk indicates significant difference from values of control at P < 0.05 (*) P < 0.01(**) by t-test

Plant CAMTAs were first identified as ethylene-induced CaM-binding proteins [17, 18]. Nonetheless, the role of ethylene in fruit ripening is well established [4]. Thus, it was interesting to study the effect of ethylene on *DzCAMTA*s during fruit ripening. The transcript abundance of *DzCAMTA3* and *DzCAMTA8* (the two highly expressed putative ripening-associated *DzCAMTA*s) was further investigated under three ripening treatments: natural ripening, ethephon-induced ripening, and 1-MCP-delayed ripening. The expression levels of *DzCAMTA3* and *DzCAMTA8* were significantly increased upon ethephon treatment, but declined drastically upon 1-MCP treatment in comparison to the natural ripening process (Fig. 7A and B). This observation provides strong evidence for the ripening-associated roles of these two *DzCAMTA*s. Furthermore, during fruit ripening, a cross-wired mesh of interactions between ethylene and auxin exists [67]. Auxin levels had been reported to be significantly altered during the ripening process in durian ‘Monthong’, thus highlighting the possible ripening-associated role of auxin [68]. This prompted us to further investigate whether there is any correlation between auxin levels and ripening-associated *DzCAMTA*s *(DzCAMTA3* and *DzCAMTA8*). *DzCAMTA3* and *DzCAMTA8* expression dramatically declined in a time-dependent manner after treatment with 40 μM exogenous auxin (Fig. 7C and D).

### Co-expression network analysis of *DzCAMTA3* and *DzCAMTA8* during post- harvest ripening

**Fig. 8:**
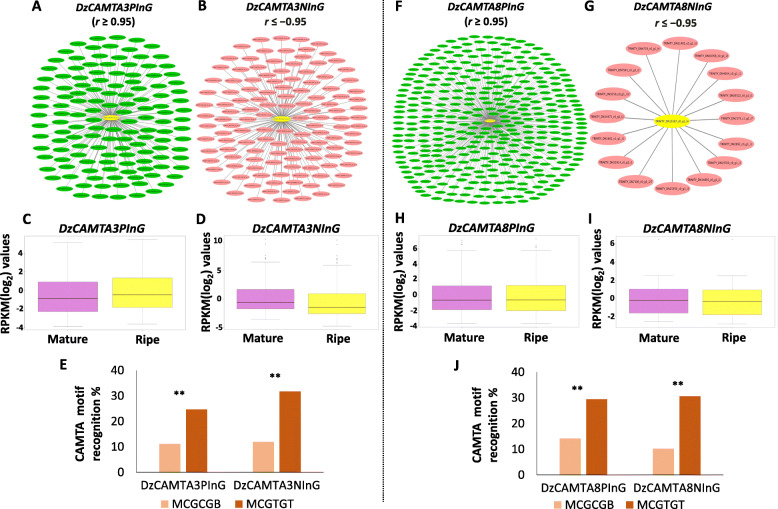
network analysis of *DzCAMTA3* and *DzCAMTA8*. **(A)** Gene co-expression network for *DzCAMTA3PinG.* **(B)** Gene co-expression network for *DzCAMTA3NinG.* **(C)** Box plot showing variation in expression level of *DzCAMTA3PinG* at mature and ripe stages of *Durio Zibethinus* pulp. **(D)** Box plot showing variation in expression level of *DzCAMTA3NinG* at mature and ripe stages of *Durio Zibethinus* pulp. **(E)** The frequency of CAMTA recognition motif (MCGCGB/MCGTGT) in *DzCAMTA3PinG* and *DzCAMTA3NinG.* The asterisks indicate significant differences (Fisher’s exact test, P < 0.05). **(F)** Gene co-expression network for *DzCAMTA8PinG.* **(G)** Gene co-expression network for *DzCAMTA8NinG.* **(H)** Box plot showing variation in expression level of *DzCAMTA8PinG* at mature and ripe stages of *Durio Zibethinus* pulp. **(I)** Box plot showing variation in expression level of *DzCAMTA8NinG* at mature and ripe stages of *Durio Zibethinus* pulp. **(J)** The frequency of CAMTA recognition motif (MCGCGB/MCGTGT) in *DzCAMTA8PinG* and *DzCAMTA8NinG.* The asterisks indicate significant differences (Fisher’s exact test, P < 0.05)

Significant RNA-sequencing (RNA-seq) data for the mature and ripe stages of durian ‘Monthong’ is publicly available. The variation in the expression of the 10 *DzCAMTAs* at mature and ripe stages were assessed using a box plot (Additional file 5- Fig. A and B). The RNA-seq data also confirmed the high expression of *DzCAMTA3* and *DzCAMTA8* during post-harvest ripening (Additional file 5- Fig. C). To identify the genes that are co-expressed with *DzCAMTA3* and *DzCAMTA8* during post-harvest ripening*,* we analyzed the RNA-seq data of durian ‘Monthong’ at the mature and ripe stages. The expression values were used in Cytoscape version 3.8.0 to identify the genes that are co-expressed with *DzCAMTA3* and *DzCAMTA8*. A total of 134 *DzCAMTA3* positively interacting genes (*DzCAMTA3PinG*) (r ≥ 0.95) (Fig. 8A and Additional file 1- Table S7) and 126 *DzCAMTA3* negatively interacting genes (*DzCAMTA3NinG*) (r ≤ − 0.95) (Fig. 8B and Additional file 1- Table S8) were identified. Similarly, 361 *DzCAMTA8* positively interacting genes (*DzCAMTA8PinG*) (r ≥ 0.95) (Fig. 8F and Additional file 1- Table S9) and 16 *DzCAMTA8* negatively interacting genes (*DzCAMTA8NinG*) (r ≤ − 0.95) (Fig. 8G and Additional file 1- Table S10) were identified. Next, the cumulative expression of *DzCAMTA3PinG*, *DzCAMTA3NinG*, *DzCAMTA8PinG*, and *DzCAMTA8NinG* (or so called *DzCAMTA3* and *DzCAMTA8* interacting genes) were assessed. *DzCAMTA3PinG* showed significantly higher cumulative expression at the ripe stage than at the mature stage (Fig. 8C), whereas the cumulative expression of *DzCAMTA3NinG* was lower at the ripe stage (Fig. 8D). In contrast, for *DzCAMTA8PinG* (Fig. 8H) and *DzCAMTA8NinG* (Fig. 8I), the cumulative expression remained unaltered from the mature to the ripe stage. Furthermore, the promoter regions (1000 bp upstream of the transcription start site) of *DzCAMTA3PinG* (134), *DzCAMTA3NinG* (126), *DzCAMTA8PinG* (361), and *DzCAMTA8NinG* (16) were scanned to identify the conserved CAMTA recognition motifs (MCGCGB/MCGTGT) (Fig. 8E and J). Interestingly, the frequency of occurrence of MCGTGT was higher than that of MCGCGB in all interacting genes. This is because the MCGTGT core motif has an overlapping binding site with the abscisic acid responsive element (ABRE) [41, 69]. It is thus conclusive to say that the presence of CAMTA recognition motifs in the promoter regions of *DzCAMTA3PinG*, *DzCAMTA3NinG*, *DzCAMTA8PinG*, and *DzCAMTA8NinG* might result in their functional regulation by *DzCAMTA3* and *DzCAMTA8.*

### Pathway and gene ontology analysis of genes co-expressed with *DzCAMTA3* and *DzCAMTA8*

*DzCAMTA3PinG*, *DzCAMTA3NinG*, *DzCAMTA8PinG*, and *DzCAMTA8NinG* were subjected to MapMan visualization and statistical analysis tool to identify the major pathways regulated by the *DzCAMTA3* and *DzCAMTA8* co-expressed genes during post-harvest fruit ripening. The MapMan analysis revealed that several *DzCAMTA3PinG* and *DzCAMTA8PinG* belonged to TFs representing AP2/ERF, DREB, ARF, SBP, bHLH, MYB, HUA2, WRKY, NAC, HSF, and bHLH families, which have been well documented to be involved in the regulation of fruit ripening processes (Additional file 6- Fig. A). Internal plant rhythms, such as the circadian clock, have been shown to affect post-harvest ripening with respect to certain plant metabolites [70]. Several *DzCAMTA3PinG* and *DzCAMTA8PinG* were found to regulate circadian clock systems, secondary metabolism, and solute transport (Additional file 6- Fig. B, C, and D). As already discussed, CAMTAs are Ca^2+^/CaM-regulated TFs that have long been linked to phytohormones, such as ABA, ethylene, and auxins [14]. Coherently, *DzCAMTA3PinG* and *DzCAMTA8PinG* were linked with positive regulation of ABA, ethylene, and auxins (Additional file 6- Fig. E). Moreover, positive regulation of CDPK, CAMK, and MAPKs were also identified in *DzCAMTA3PinG* and *DzCAMTA8PinG* (Additional file 6- Fig. F). Carbohydrate metabolism and cell wall structural changes during post-harvest ripening have long been the subject of study [71, 72]. It was interesting to note that *DzCAMTA8PinG* were involved in carbohydrate metabolism as well as cell wall and its precursor synthesis (Additional file 6- Fig. G and H). Additionally, *DzCAMTA8PinG* were also involved in external stimulus response (Additional file 6- Fig. I), nutrient uptake (Additional file 6- Fig. J), and photosynthesis (Additional file 6- Fig. K). In contrast, *DzCAMTA3NinG* and *DzCAMTA8NinG* were involved in fewer functional processes, including co-enzyme metabolism, solute transport by the OPT family, protein modification (TLK, CMGC, and CAMK protein kinase families), and targeting of TF families (C2H2 and FAR1). Furthermore, GO enrichment analysis was performed to gain insights into the biological processes, molecular functions, and cellular components governed by *DzCAMTA3* and *DzCAMTA8* co-expressed genes. Taking into account *DzCAMTA3* interacting genes, several GO categories related to biotic and abiotic stress, fruit development, seed development, and ripening were significantly affected. This included drought recovery, root hair cell tip growth, pollen tube growth, cell tip growth, pollen tube development, response to UV, pollination, embryo development ending in seed dormancy, embryo development, seed development, inositol 3-kinase activity, carbohydrate kinase activity, 1-aminocyclopropane-1-carboxylate biosynthetic process, and clathrin-coated endocytic vesicle membrane (Additional file 7). The GO categories significantly affected by *DzCAMTA8* interacting genes included major biotic stress-related and fruit ripening-related categories such as MAP kinase activity, defense response, response to ethylene, response to salicylic acid, jasmonic acid metabolic process, regulation of ABA signaling pathway, immune system process, response to wounding, response to hydrogen peroxide, and vegetative to reproductive phase transition of meristem. One interesting GO category associated with *DzCAMTA8* interacting genes was CaM binding (molecular function), validating the role of *DzCAMTA8* as a CaM-binding TF (Additional file 8). Additionally, the GO categories for the 10 *DzCAMTA* genes were also assessed (Additional file 9). As expected, CaM binding was the highest enriched GO in the molecular function category for *DzCAMTA*s (Additional file 10).

## Discussion

The recent availability of the *Durio zibethinus* draft genome [73] allowed us to perform a comprehensive analysis of the CAMTA gene family in durian with respect to post-harvest ripening. The implication of CAMTA genes in tomato fruit ripening [27] further prompted us to study the role of *DzCAMTA*s in same direction. We undertook a comprehensive genome-wide characterization and expression analysis to establish the role of DzCAMTA TFs in post-harvest fruit ripening. The present study identified 10 putative *DzCAMTA* genes in the durian genome (Table 1). Domain organization analysis of DzCAMTAs revealed that except DzCAMTA10 (non-TIG CAMTA), all other DzCAMTAs possessed the conventional CAMTA domains (Fig. 1A). Several other plant species, such as *Arabidopsis lyrata, Gossypium raimondii, Gossypium hirsutum, Gossypium arboretum, and Capsella rubella* lack the TIG domain in their reported CAMTAs [24, 52]. It is hypothesized that during evolution, instead of complete deletion of this domain in CAMTAs, mutation of some vital amino acids probably occurred [52]. Thus, apparently the generation of non-TIG CAMTAs led to the expansion of the CAMTA gene family in durian. All the predicted DzCAMTAs were long proteins comparable to their counterparts in other plant species such as *Arabidopsis thaliana* [25] and *Gossypium* [24]. All DzCAMTAs localized to the nucleus with a high reliability index (Table 1), had similar exon-intron organization, and motif structures (Fig. 1C, D and Additional file 1- Table S3). The phylogenetic relationship of DzCAMTAs with other plant species indicated that the major groups contained orthologs from *Arabidopsis thaliana*, *Solanum lycopersicum, Theobroma cacao, and Gossypium hirsutum.* This proposes a similar function of DzCAMTAs with other members clustered together. For example, DzCAMTA3 grouped together with *Solanum lycopersicum* Solyc01g105230 (*SlSR1L*) (Fig. 2). *SlSR1L* is reported during tomato enlargement and ripening [27], which led us to hypothesize the similar function of DzCAMTA3 in durian fruit ripening. Similarly, DzCAMTA8 grouped with AtCAMTA3 (Fig. 2). The involvement of AtCAMTA3 has been shown to regulate biotic stress responses [22, 43, 44]. Coherently, biotic phenomenon also affects fruit ripening and senescence [74].

Hoarding evidences suggest that the expansion of gene families due to gene duplication events is one of the pivotal evolutionary mechanisms contributing to functional diversification and speciation [75]. In concurrence, the localization of paralogous durian *CAMTA* gene pairs on different scaffolds are indicative of segmental duplication events (Fig. 3). Moreover, the predominance of purifying selection in paralogous durian *CAMTA* gene pairs implies the selective removal of deleterious alleles. Loss-of-function mutations are possibly obliterated by purifying selection, thus fixing the new duplicate gene and ameliorating functional alleles at both duplicate loci [76]. Interestingly, *DzCAMTA1* and *DzCAMTA3* (the paralogous gene pair) showed opposite expression profiles during post-harvest ripening (Fig. 6). This substantiates the fact that this paralogous gene pair might have different functions in durian. Furthermore, the duplication time of *GhCAMTA*s (*Gossypium* is the closest relative of durian) is 13.04 MYA [24], whereas for *DzCAMTA*s is 14.64 to 17.95 MYA. This implies that the duplication events in the durian CAMTA families were more ancient than polyploid formation. Probably, these duplications may be responsible for the unique functions of CAMTAs in durian, i.e., post-harvest ripening.

Ca^2+^ is an important second messenger that mediates responses to external stimuli and hormonal fluctuations via Ca^2+^ sensors, such as CaM [14, 77, 78]. The role of Ca^2+^ is further extended in fruit ripening via the intervention of cross-wired mesh of regulatory networks [27, 77]. Much alike all the other CAMTAs that have been characterized, the 10 DzCAMTAs encoded a type of Ca^2+^/CaM-binding TF (Fig. 4). It has been well-established that Ca^2+^/CaM binding to CAMTAs is critical for its in-vivo functions [18, 79]. Therefore, it can be reasonably speculated that one of the mechanisms by which Ca^2+^ mediates fruit ripening is the formation of a Ca^2+^/CaM complex to activate DzCAMTAs. DzCAMTAs might in-turn regulate the expression of downstream genes involved in fruit ripening process. Thus, DzCAMTAs probably act as co-ordinators for several cross-wired signaling pathways implicated in fruit development and ripening (Fig. 7). Furthermore, the interaction of CaM with CaMBD occurs in a Ca^2+^-dependent manner. In contrast, the IQ motif interacts with CaM in a Ca^2+^-independent manner [19, 20, 25, 27, 52, 79]. Interestingly, all the putative DzCAMTAs possessed a conserved CaMBD adjacent to the IQ motif (Fig. 1A and Fig. 4). This might imply that the interaction of CaM with DzCAMTAs can occur either in a Ca^2+^-dependent manner or in a Ca^2+^-independent manner. Moreover, the invariable presence of certain important development-related (MYB, HD-Zip1, and GCN-4 motif) and phytohormonal-related (ABRE, AuxRR-core, CGTCA, and ERE) *cis-*acting elements in the promoter region of *DzCAMTA*s strengthened their involvement in fruit development and ripening (Fig. 5). Conceivably, the occurrence of these motifs in the promoter regions of *DzCAMTA*s is indicative of the plausible molecular regulation which can be further confirmed through sequencing.

Gene expression analysis was performed to validate the involvement of *DzCAMTA*s in the post-harvest ripening of durian (Fig. 6, Additional file 4, Additional file 5). Gene expression analysis identified *DzCAMTA3* and *DzCAMTA8* as the highest expressing *DzCAMTA*s from mature to ripe stage (post-harvest ripening) (Fig. 6, Additional file 4, Additional file 5). Consequently, *DzCAMTA3* and *DzCAMTA8* were contemplated for their involvement in post-harvest ripening of durian. As previously discussed, CAMTA TF family was initially identified as an ethylene-induced CaM-binding protein [17]. Ethylene acts as the main phytohormone in regulating the ripening of climacteric fruits. Ethylene regulates fruit ripening via a complex network of interacting signaling pathways and ripening-associated developmental factors. Thus, it was interesting to study the transcript abundance of these putative ripening-associated *DzCAMTA*s under natural ripening conditions and ethylene induced/delayed ripening conditions. The expression levels of *DzCAMTA3* and *DzCAMTA8* were enhanced under ethephon treatment, while suppressed under 1-MCP treatment (Fig. 7A and B). The effect of ethylene released by ethephon [80] and the ethylene inhibition by 1-MCP [81, 82] are well documented. Therefore, the up-regulation of *DzCAMTA3* and *DzCAMTA8* by ethephon and down-regulation by 1-MCP affirms their involvement in ethylene-induced fruit ripening. Additionally, the transcript abundance of these ethylene-induced *DzCAMTA*s increased during post-harvest ripening (Fig. 6, Additional file 4, Additional file 5). Therefore, *DzCAMTA3* and *DzCAMTA8* are speculated to function as ethylene-induced transcriptional activators of ripening. Moreover, as mentioned above, in a similar study, the CAMTA TF family member from *Solanum lycopersicum* (SlSR1L) has been reported to be ethylene-induced (phylogenetically grouped together with *DzCAMTA3*) and acts as a transcriptional activator of ripening [27]. This further asserts the observations made in the present study. Similarly, transcriptional regulation of auxin biosynthetic genes has been previously shown to mediate post-harvest ripening of the Monthong cultivar by the joint action of *DzARF2A* and *DzDof2.2* [68, 83]. In agreement with previous reports, *DzCAMTA3* and *DzCAMTA8* (Fig. 7C and D) expression dramatically declined in a time-dependent manner upon exogenous auxin treatment. This indicated a positive correlation of ethylene with *DzCAMTA3* and *DzCAMTA8,* while a negative correlation of auxin with *DzCAMTA3* and *DzCAMTA8.* Consequently, *DzCAMTA3* and *DzCAMTA8* synergistically crosstalk with ethylene during the post-harvest ripening of the durian fruit. In contrast, *DzCAMTA3* and *DzCAMTA8* antagonistically with auxin could mediate the post-harvest ripening of durian fruit.

The co-expression analysis of *DzCAMTA3* and *DzCAMTA8* suggested that they may be involved in regulating complex gene networks underlying post-harvest ripening of climacteric fruits (Fig. 8A, B, F, G, Additional file 1- Table S7, S8, S9, and S10). *DzCAMTA3PinG*, *DzCAMTA3NinG*, *DzCAMTA8PinG*, and *DzCAMTA8NinG* belonged to UDP-sugar pyrophosphorylase [84], heat shock TF [85], E3 ubiquitin ligase [86], GATA zinc finger TF [87], protein kinase [88], NADH dehydrogenase [89], 1-amino-cyclopropane-1-carboxylate synthase [90], TIFY [91], cytochrome P450 [92], cyclin dependent kinase [93], ethylene responsive factor [2], homeodomain subfamily [94], WRKY [95], MYB [96], auxin-responsive factor AUX/IAA [83], MAP kinase [97], squamosa promoter binding protein [98], C2H2 zinc finger protein [99], and AP2/B3 TF [100] protein families, which have been previously reported to play significant roles in fruit ripening. The presence of 1-aminocyclopropane-1-carboxylic acid (ACC) synthase enzyme (catalyzes the synthesis of ACC-precursor of ethylene) in *DzCAMTA3NinG* was a very interesting observation in the present study with respect to fruit ripening (Additional file 1- Table S8). Our results suggest that CAMTAs can act as both positive and negative regulators of gene expression during fruit ripening. As such, *AtCAMTA3* acts as a positive regulator of the CBF2 regulon [42], and also acts as a negative regulator of SA-mediated immune responses [44, 101]. Thus, the presence of ACC synthase in *DzCAMTA3NinG* was not surprising. In a similar vein, the presence of ACC oxidase (catalyzes the conversion of ACC to ethylene) in *DzCAMTA8PinG* was equally interesting (Additional file 1- Table S9). The positive co-expression of DzCAMTA8 with ACC oxidase suggests the involvement of this TF in regulating the intricate signaling network of fruit ripening process. The enrichment of CAMTA recognition motifs (MCGCGB/MCGTGT) in the promoter sequences of *DzCAMTA3*-and *DzCAMTA8* interacting genes also supports the assertions made in the present study (Fig. 8E and J). Thus, *DzCAMTA3* and *DzCAMTA8* via a complex regulatory mesh could regulate fruit ripening through *DzCAMTA3PinG*, *DzCAMTA3NinG*, *DzCAMTA8PinG*, and *DzCAMTA8NinG.* One intriguing observation was the significantly greater frequency of occurrence of the MCGTGT motif than MCGCGB in *DzCAMTA3* and *DzCAMTA8* interacting genes*.* A plausible explanation for this observation could be that the MCGTGT core motif has an overlapping binding site with ABRE, which is also recognized by bZIP proteins [41, 69], resulting in a greater frequency. It is also important to mention that plant CAMTAs regulate various stress, phytohormonal, and ROS responses in plants [14]. ROS response, phytohormonal regulation, and redox state are equally imperative for fruit ripening [102–104]. The identification of pathways belonging to these categories in *DzCAMTA3PinG*, *DzCAMTA3NinG*, *DzCAMTA8PinG*, and *DzCAMTA8NinG* supports the importance of DzCAMTA3 and DzCAMTA8 in fruit ripening (Additional file 6- Fig. A, C and E). Cell wall [105] and carbohydrate metabolism [71] are often linked with fruit ripening, and the regulation of these pathways by *DzCAMTA3* and *DzCAMTA8* interacting genes further emphasize their involvement in fruit ripening (Additional file 6- Fig. G and H). Finally, the enrichment of certain important GO terms related to ACC biosynthetic process, amino peptidase activity, fructose 2,6-bisphosphate metabolic process, ethylene response, inositol -3 kinase, embryo development, CaM binding, MAPK cascade, etc. in *DzCAMTA3* and *DzCAMTA8* interacting genes substantiate their role in the fruit ripening process (Additional file 7 and Additional file 8).

## Conclusion

Taken together, our results suggest that *DzCAMTA3* and *DzCAMTA8* are the key components of the regulatory network underlying post-harvest ripening in durian (Additional file 11). The present study also represents another layer of intricate gene regulatory network involved in fruit ripening. The study further emphasizes the fact that the ripening process heavily relies on the interplay between different TFs and hormones. The findings from this study can be applied to the development of molecular markers for producing new durian varieties with prolonged shelf life. Nonetheless, rigorous analysis unraveling the underpinning molecular mechanisms of DzCAMTAs in fruits will provide greater insights into their ripening-associated roles, which remain elusive and a subject for further study.

## Methods

### Plant materials and exogenous treatments

Durian cultivar Monthong (*D. zibethinus* Murr. ‘Monthong’) fruits were collected from a commercial orchard at Trat province of Thailand. Durian fruits of almost similar weight (~ 3–4 kg) and size were harvested at the immature and mature stages. The immature stage for Monthong was at 85 days after anthesis (DAA) [50, 68, 83], while the mature stage was at 105 DAA [50, 68, 83]. Immature durian fruit samples and some mature durian fruit samples were peeled immediately after harvesting. The remaining mature durian fruit samples were stored at room temperature (RT ~ 30 °C) until peeling. In this study, durian fruit samples were obtained at five different stages: immature 1, immature 2, mature, mid ripe, and ripe. For mature, mid ripe, and ripe samples, fruits were harvested at the mature stage, kept at RT for one, three, and five days and then peeled, respectively. After peeling, two central pulps from each fruit were collected according to the protocol described in [50]. To ensure that the durian fruit samples were compared at the same ripening stage, the first pulp was collected along with a seed, and fruit firmness was measured using a texture analyzer (TA-XT2i; Stable Micro Systems, Godalming, UK) according to the protocol described in [68]. A puncture test was performed at five random points in each pulp at a test speed of 2 mm/s and a test distance of 5 mm using a 6-mm probe. Midripe pulps had a mean ± standard deviation (SD) of 3.4 ± 0.81 N, whereas ripe pulps had a mean ± SD of 1.55 ± 0.45 N. After this assessment, the pulp was collected without seeds. It was immediately snap frozen in liquid nitrogen and stored at − 70 °C for RNA preparation.

Three different ripening treatments (natural ripening, ethephon-induced, and 1-methylcyclopropene (1-MCP)-delayed ripening) were compared to study the effect of ethylene on *DzCAMTA3* and *DzCAMTA8* during post-harvest ripening. Ethephon (48% 2-chloroethylphosphonic acid; Alpha Agro Tech Co., Ltd., Thailand) solution was applied to the upper area of each fruit stalk (mature durian samples). For treating the mature durian samples with 1-MCP, each fruit was placed in a 20 L closed chamber. To this chamber one tablet of 1-MCP (0.19% 1-MCP tablet; BioLene Co., Ltd., China) was placed in a beaker. Five milliliters water was added to the beaker containing 1-MCP tablet to produce gaseous 1-MCP. The chamber was immediately closed at RT for 12 h. Control samples were treated in the same manner at the same temperature and for the same period of time without 1-MCP. After 12 h, the control and 1-MCP–treated samples were kept at RT for ripening and collected the same day when ethephon treated durian samples were collected. The samples were then peeled and processed as described previously. Three independent biological replicates of each sample type were used in this study. Each biological replicate represented one durian fruit, which was harvested from a separate tree.

For exogenous auxin application, young leaves of *D. zibethinus* Murr. ‘Monthong’ were soaked in 40 μM of indole-3-acetic acid (IAA) (Duchefa Biochemie, the Netherlands) for 30, 60, and 120 min. For the control samples, the leaves were soaked in distilled water without IAA. Immediately after the completion of the treatment, the leaves were snap frozen in liquid nitrogen and stored at − 70 °C for RNA preparation.

### Identification of *CAMTA* gene family in *Durio zibethinus*

The whole-genome protein sequence of *Durio zibethinus* was downloaded from the National Center for Biotechnology Information (NCBI) (https://www.ncbi.nlm.nih.gov/). A total of 1343 CAMTA protein domain sequences from 166 plant species were downloaded from the Plant Transcription Factor Database (http://planttfdb.gao-lab.org/index.php) [106]. These two sets of data were used to construct a Hidden Markov Model (HMM) profile. Subsequently, the HMM profile of the conserved CAMTA domains (CG-1, CaMBD, TIG domain, ankyrin repeats, and IQ) was utilized as a query in the HMMER (V3.0) software to identify the putative *Durio zibethinus* CAMTAs. The redundant and partial sequences were removed and the *Dz*CAMTAs were named numerically.

### Domain organization, gene structure and conserved motif analysis of DzCAMTAs

All *Dz*CAMTAs were subjected to the Pfam database (http://pfam.xfam.org/), NCBI Conserved Domain Database (https://www.ncbi.nlm.nih.gov/cdd/), and InterPro database (https://www.ebi.ac.uk/interpro/search/sequence/) to confirm the presence of conserved CAMTA domains. Nuclear localization signal was queried using the Motif Scan (http://myhits.isb-sib.ch/cgi-bin/motif_scan) and NLS Mapper (http://nls-mapper.iab.keio.ac.jp/cgi-bin/NLS_Mapper_form.cgi). CaMBD was mapped using the Calmodulin Target Database (http://calcium.uhnres.utoronto.ca/ctdb/ctdb/home.html). Finally, a schematic representation of the *Dz*CAMTAs functional domains was constructed using Illustrator for Biological Sequences software (http://ibs.biocuckoo.org/) [107]. The gene structures of *DzCAMTA*s were analyzed by querying *DzCAMTA* coding sequences with their corresponding genomic sequences on the Gene Structure Display Server (GSDS) (http://gsds.cbi.pku.edu.cn/) [108]. Conserved protein motifs were determined using the Multiple Em for Motif Elicitation (MEME) tool (https://meme-suite.org/meme/tools/meme) [109] with the following parameters: width from “6 to 300”, “zero” or “one” per sequence, and maximum number of motifs to search “20”. The motifs were annotated using the InterproScan software.

### Computation of physicochemical properties and subcellular localization of DzCAMTAs

The physicochemical properties of the DzCAMTA proteins were computed using ProtParam (http://web.expasy.org/protparam/). The subcellular localization of DzCAMTA proteins was determined by CELLO v.2.5, (http://cello.life.nctu.edu.tw/).

### Multiple sequence alignment (MSA) and phylogenetic tree construction of DzCAMTA proteins

CAMTA protein sequences from four different plant species were downloaded from the Plant Transcription Factor Database (http://planttfdb.gao-lab.org/index.php) [106]. MSA was performed using the Clustal Omega program (https://www.ebi.ac.uk/Tools/msa/clustalo/) with default parameters. MEGA X software [110] was used to construct a maximum likelihood (ML) phylogenetic tree. The parameters employed included pairwise gap deletion, the JTT model, and 1000 iterations of bootstrap values.

### Scaffold gene physical position and gene duplication analysis

The physical locations of all 10 DzCAMTAs were obtained using a BLASTN search. The *DzCAMTA* genes were mapped onto the related scaffolds using Mapinspect software. The paralogous durian *CAMTA* gene pairs were identified by reciprocal blast analysis with an e-value < 10^− 5^. The parameters used were query coverage > 70% of the longer sequence and percentage identity > 70% [111]. Ka/Ks analysis was performed using PAL2NAL [112]. The Ks values attained for each gene pair were converted into divergence time in million years ago (MYA), assuming a rate of 6.1 × 10^− 9^ substitutions per site per year for eudicots [75, 113]. The divergence time (T) was estimated as T = Ks/2λ (λ = 6.1 × 10^− 9^) [114]. Further, a Ka/Ks ratio < 1 indicates negative (purifying selection), > 1 indicates positive selection, and = 1 indicates neutral selection [115].

### RNA-seq analysis, generation of heat map and box plot

Illumina sequencing reads from RNA-seq of durian (Monthong cultivar) were retrieved from the public repository Sequence Read Archive (SRA) at NCBI (project number: PRJNA683229) [116]. The method in [68] was used to obtain the *de-novo* assembled transcriptome. The input reads were aligned to the *de-novo* assembled transcriptome using the abundance estimation tool of the Trinity package to generate raw counts for each contig. Raw read counts were merged into a single read count matrix. The single read count matrix was normalized to obtain a trimmed mean of the M-values (TMM) normalized matrix. Normalized total read counts were used to generate a heatmap. The heatmap was drawn using MetaboAnalyst 5.0 [117], an R-based public database. The parameters used for heatmap generation were as follows: normalization by a pooled sample from the group, log_2_ transformed, and autoscaled. Similarly, box plots were drawn using the ggplot2 (https://cran.r-project.org/web/packages/ggplot2/) package in R version 4.0.3 GUI 1.73.

### Co-expression network and promoter analysis of *DzCAMTA3* and *DzCAMTA8* interacting genes

Expression values were used to generate a gene co-expression network. The “Expression Correlation Networks” (http://apps.cytoscape.org/apps/expressioncorrelation) plugin of Cytoscape version 3.8.0. was used to calculate positive Pearson correlation (default r ≥ 0.95) and “anti-correlation” or negative Pearson correlation (default r ≤ − 0.95) in the interacting members of a network. Network visualization was performed by applying a force-directed layout in Cytoscape. The nodes (circles) represent genes and edges (straight lines) represent significant interactions between the expression levels of genes from the mature to the ripe stage (gene correlation network). The promoter sequences with the required length of 1000 bp for *DzCAMTA3* and *DzCAMTA8* interacting genes were fetched by running a shell script on a multi-fasta file with genomic sequences and a GFF file for the multi-fasta file available at NCBI. The other dependencies (required packages) included Samtools (http://www.htslib.org/) and bedtools (https://bedtools.readthedocs.io/en/latest/). Further, another in-house python script was run to identify the MCGCGB and MCGTGT motifs in the promoter regions (complement and reverse complement) of *DzCAMTA3* and *DzCAMTA8* interacting genes.

### Promoter *cis-*acting element analysis of *DzCAMTAs*

DNA sequences 1000 bp upstream of the transcription start site for *DzCAMTA*s were retrieved from NCBI. These sequences were queried using PlantCARE [53] (http://bioinformatics.psb.ugent.be/webtools/plantcare/html/) and PLACE database (https://www.dna.affrc.go.jp/PLACE/?action=newplace) [54] for the identification of *cis*-acting elements.

### Pathway and gene ontology analysis of *DzCAMTA3* and *DzCAMTA8* interacting genes

Pathway analysis of *DzCAMTA3PinG*, *DzCAMTA3NinG*, *DzCAMTA8PinG*, and *DzCAMTA8NinG* was performed using MapMan software (http://www.gabipd.org/projects/MapMan/) [118]. Multiple correction tests (Benjamini Hochberg) with FDR < 0.05, were used to fetch significant functional categories or metabolic pathways. Furthermore, the Blast2GO feature of OmicsBox (https://www.blast2go.com/) was utilized to identify significant Gene Ontology (GO) terms with respect to three aspects: biological processes, molecular functions, and cellular components.

### RNA isolation and real-time quantitative RT-PCR

Total RNA was isolated from durian pulp. The isolated RNA was purified using PureLink Plant RNA Reagent (Thermo Fisher Scientific, MA, US) according to the manufacturer’s protocol. Genomic DNA contamination was removed by treating the purified RNA with DNase I (Thermo Fisher Scientific, MA, US). RNA integrity was assessed by agarose gel electrophoresis and an Eppendorf BioPhotometer D30 with A_260_/A_280_ (ratio between 1.8 to 2.0) and A_260_/A_230_ (ratios between 2.0 to 2.2). One microgram of DNase I-treated RNA was used to synthesize first-strand cDNA. cDNA was prepared using the RevertAid First Strand cDNA Synthesis Kit (Thermo Fisher Scientific, MA, US) according to the manufacturer’s instructions. The elongation factor 1 alpha (*EF-1α*) gene was used as an internal control. The qRT-PCR reaction was performed in a 20-μL reaction volume by adding 5 pmol of gene-specific primers, 1 μL cDNA of each sample to 2X Luna Universal qPCR Master Mix (New England Biolabs, MA, USA). PCR was carried out on a CFX95 Real-time System (Bio-Rad Laboratories Inc., CA, USA) with the following program: initial denaturation at 95 °C for 1 min, followed by 40–45 cycles of denaturation at 95 °C for 15 s, and annealing/extension at 60 °C for 30 s. Melt curve analysis to examine the specificity of primers was performed from 60 °C to 95 °C in increments of 0.5 °C. Transcript abundance was calculated as a comparative fold change following the 2^−ΔΔct^ quantitative method [119]. Three independent biological replicates and three technical replicates were used for the statistical analysis.

## Supplementary Information

**Additional file 1: Table S1.** All identified durian CAMTAs. **Table S2.** Protein sequence of DzCAMTAs. **Table S3.** List of 20 conserved protein motif sequences and their annotation in DzCAMTAs. **Table S4.** List of CAMTA genes from five species used for the phylogenetic analysis of DzCAMTAs. **Table S5.** The Ka/Ks ratios and date of duplication for duplicate CAMTA genes in Durio zibethinus. **Table S6.** List of predicted cis-acting elements in the promoter regions of DzCAMTAs. **Table S7.** List of positively interacting genes (Pearson correlation coefficient r ≥ 0.95) with DzCAMTA3. **Table S8.** List of negatively interacting genes (Pearson correlation coefficient r ≤ -0.95) with DzCAMTA3. **Table S9.** List of positively interacting genes (Pearson correlation coefficient r ≥ 0.95) with DzCAMTA8. **Table S10.** List of negatively interacting genes (Pearson correlation coefficient r ≤ -0.95) with DzCAMTA8.

**Additional file 2.** Alignment of 10 putative DzCAMTAs proteins with 6 Arabidopsis CAMT As. The conserved domains associated with CAMTA proteins (CG-1, NLS, TIG domain, ankyrin repeat, IQ motif, and CaMBD) are marked below the alignment by orange line.

**Additional file 3.** Twenty enriched motifs in10 putative DzCAMTAs.

**Additional file 4. **Real time validation of 10 putative *DzCAMTA* genes in *Durio Zibethinus* pulp at five different stages (Immature1-IM1, Immature2-IM2, Mature-M, Mid-ripe-MR, and Ripe-R) during post-harvest ripening. Data are the mean ± SE of three different biological replicates and three technical replicates. Asterisk indicates significant difference from values of M at *P* < 0.05 (*) *P* < 0.01(**) by t-test.

**Additional file 5. **Expression profile of *DzCAMTA*s during post-harvest ripening. Variation in expression of 10 *DzCAMTA*s at **(A)** mature stage and **(B)** ripe stage of *Durio Zibethinus* was visualized by box plot. Each *DzCAMTA* is represented with a different color. The central line for each box plot indicates the median value. The expression values correspond to the RPKM. **(C)** Expression profiles of 10 *DzCAMTA*s in mature and ripe stages of *Durio Zibethinus* pulp. Data was sum normalized, log transformed, and auto scaled.

**Additional file 6. **Functional and GO annotation of *DzCAMTA3* and *DzCAMTA8* interacting genes. MapMan based functional classification of *DzCAMTA3PinG*, *DzCAMTA3NinG*, *DzCAMTA8PinG*, and *DzCAMTA8NinG.* **(A)** RNA biosynthesis transcriptional regulation. **(B)** Multi-process regulation. **(C)** Coenzyme metabolism, secondary metabolism and redox homeostasis. **(D)** Solute transport. **(E)** Phytohormone action. **(F)** Protein modification. **(G)** Carbohydrate metabolism. **(H)**Cell wall organization. **(I)** External stimuli response. **(J)** Nutrient uptake. **(K)** Photosynthesis. The scale represents expression values in log_2_.

**Additional file 7. **GO annotation of *DzCAMTA3PinG*, and *DzCAMTA3NinG* with respect to biological processes, molecular functions and cellular components, respectively.

**Additional file 8. **GO annotation of *DzCAMTA8PinG*, and *DzCAMTA8NinG* with respect to biological processes, molecular functions and cellular components, respectively.

**Additional file 9. **GO functional annotation for the 10 *DzCAMTAs.* The different colors represent the proportions of various GO terms.

**Additional file 10. **GO annotation of 10 *DzCAMTAs* with respect to biological processes, molecular functions and cellular components, respectively.

**Additional file 11. **Schematic representation of the role of ripening associated *DzCAMTA3*, and *DzCAMTA8* in the regulatory network of durian fruit ripening.

## Data Availability

The Illumina RNA-seq sequencing reads used in the current study are available in the public repository Sequence Read Archive (SRA) at NCBI, and the project number is PRJNA683229 (https://www.ncbi.nlm.nih.gov/Traces/study/?acc=PRJNA683229).

## Abbreviations

CAMTA: Calmodulin binding transcription activator
*DzCAMTA3PinG*: *DzCAMTA3* positively interacting genes
*DzCAMTA3NinG*: *DzCAMTA3* negatively interacting genes
*DzCAMTA8PinG*: *DzCAMTA8* positively interacting genes
*DzCAMTA8NinG*: *DzCAMTA8* negatively interacting genes
