# Supplementary material for: Comprehensive genome-wide analysis of calmodulin-binding transcription activator (CAMTA) in Durio zibethinus and identification of fruit ripening-associated DzCAMTAs
Source: BMC Genomics. 2021 Oct 14;22:743. doi: 10.1186/s12864-021-08022-1 (PMC8518175; doi:10.1186/s12864-021-08022-1)
Supplement: Supplementary file 2 — Additional file 2. Alignment of 10 putative DzCAMTAs proteins with 6 Arabidopsis CAMT As. The conserved domains associated with CAMTA proteins (CG-1, NLS, TIG domain, ankyrin repeat, IQ motif, and CaMBD) are marked below the alignment by orange line. [file 12864_2021_8022_MOESM2_ESM.pdf]

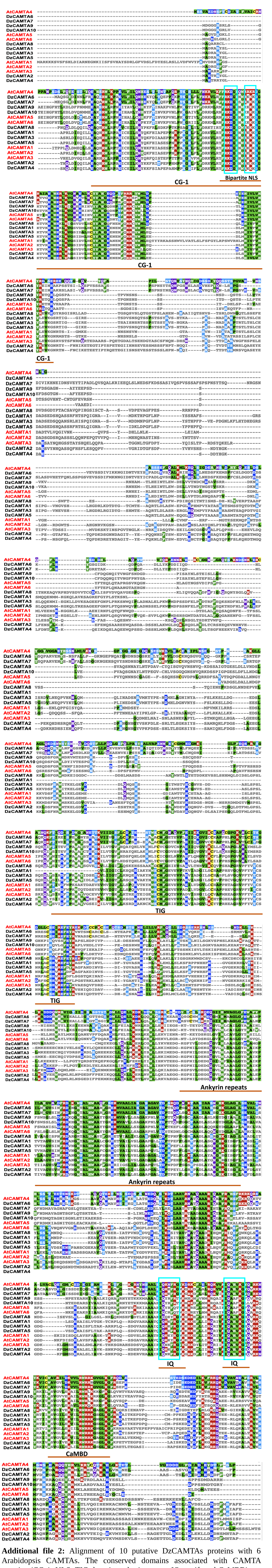

Additional file 2: Alignment of 10 putative DzCAMTAs proteins with 6 Arabidopsis CAMTAs. The conserved domains associated with CAMTA proteins (CG-1, NLS, TIG domain, ankyrin repeat, IQ motif, and CaMBD) are marked below the alignment by orange line.
