# Supplementary figures and images for: Comprehensive genome-wide analysis of calmodulin-binding transcription activator (CAMTA) in Durio zibethinus and identification of fruit ripening-associated DzCAMTAs
Source: BMC Genomics. 2021 Oct 14;22:743. doi: 10.1186/s12864-021-08022-1 (PMC8518175; doi:10.1186/s12864-021-08022-1)

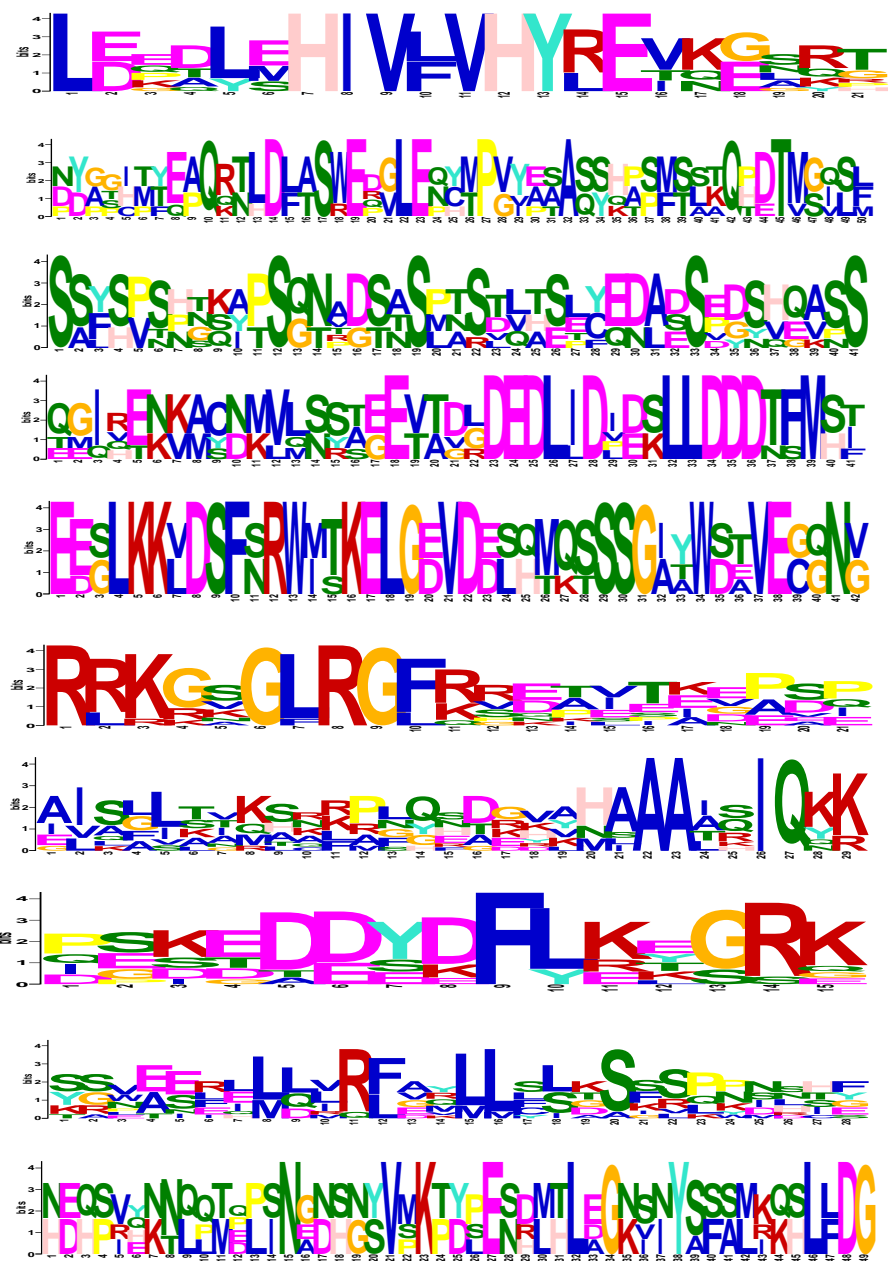

### Additional file 3: Twenty enriched motifs in 10 putative DzCAMTAs.

Supplement: Supplementary file 3 — Additional file 3. Twenty enriched motifs in10 putative DzCAMTAs. [file 12864_2021_8022_MOESM3_ESM.pdf]
