# Supplementary material for: Comprehensive genome-wide analysis of calmodulin-binding transcription activator (CAMTA) in Durio zibethinus and identification of fruit ripening-associated DzCAMTAs
Source: BMC Genomics. 2021 Oct 14;22:743. doi: 10.1186/s12864-021-08022-1 (PMC8518175; doi:10.1186/s12864-021-08022-1)
Supplement: Supplementary file 4 — Additional file 4. Real time validation of 10 putative DzCAMTA genes in Durio Zibethinus pulp at five different stages (Immature1-IM1, Immature2-IM2, Mature-M, Mid-ripe-MR, and Ripe-R) during post-harvest ripening. Data are the mean ± SE of three different biological replicates and three technical replicates. Asterisk indicates significant difference from values of M at P < 0.05 (*) P < 0.01(**) by t-test. [file 12864_2021_8022_MOESM4_ESM.pdf]

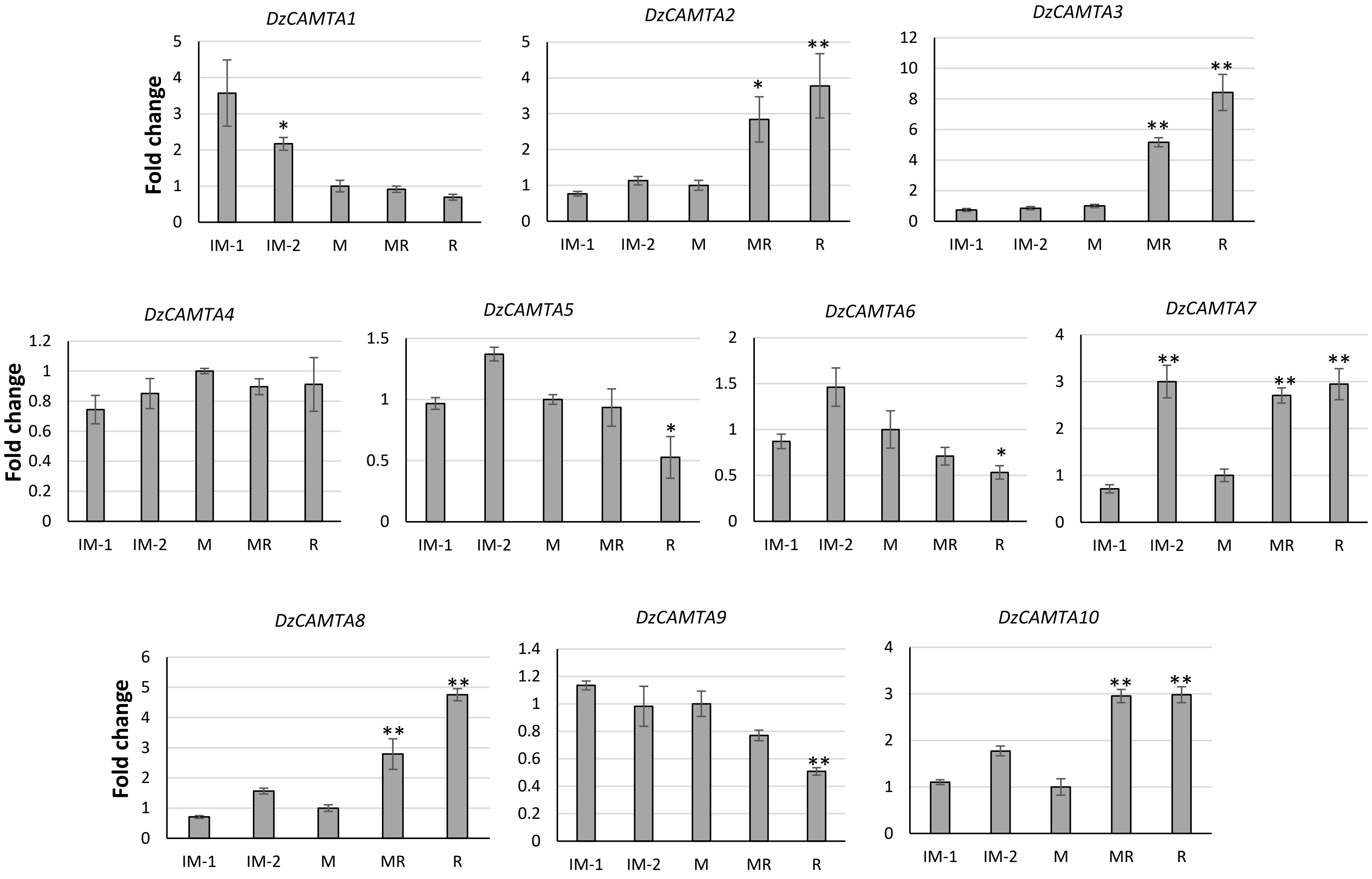

Additional file 4: Real time validation of 10 putative *DzCAMTA* genes in *Durio Zibethinus* pulp at five different stages (Immature1-IM1, Immature2-IM2, Mature-M, Mid-ripe-MR, and Ripe-R) during post-harvest ripening. Data are the mean ±SE of three different biological replicates and three technical replicates. Asterisk indicates significant difference from values of M at $P < 0.05$ (*) $P < 0.01$(**) by t-test.
