# Supplementary material for: Comprehensive genome-wide analysis of calmodulin-binding transcription activator (CAMTA) in Durio zibethinus and identification of fruit ripening-associated DzCAMTAs
Source: BMC Genomics. 2021 Oct 14;22:743. doi: 10.1186/s12864-021-08022-1 (PMC8518175; doi:10.1186/s12864-021-08022-1)
Supplement: Supplementary file 5 — Additional file 5. Expression profile of DzCAMTAs during post-harvest ripening. Variation in expression of 10 DzCAMTAs at (A) mature stage and (B) ripe stage of Durio Zibethinus was visualized by box plot. Each DzCAMTA is represented with a different color. The central line for each box plot indicates the median value. The expression values correspond to the RPKM. (C) Expression profiles of 10 DzCAMTAs in mature and ripe stages of Durio Zibethinus pulp. Data was sum normalized, log transformed, and auto scaled. [file 12864_2021_8022_MOESM5_ESM.pdf]

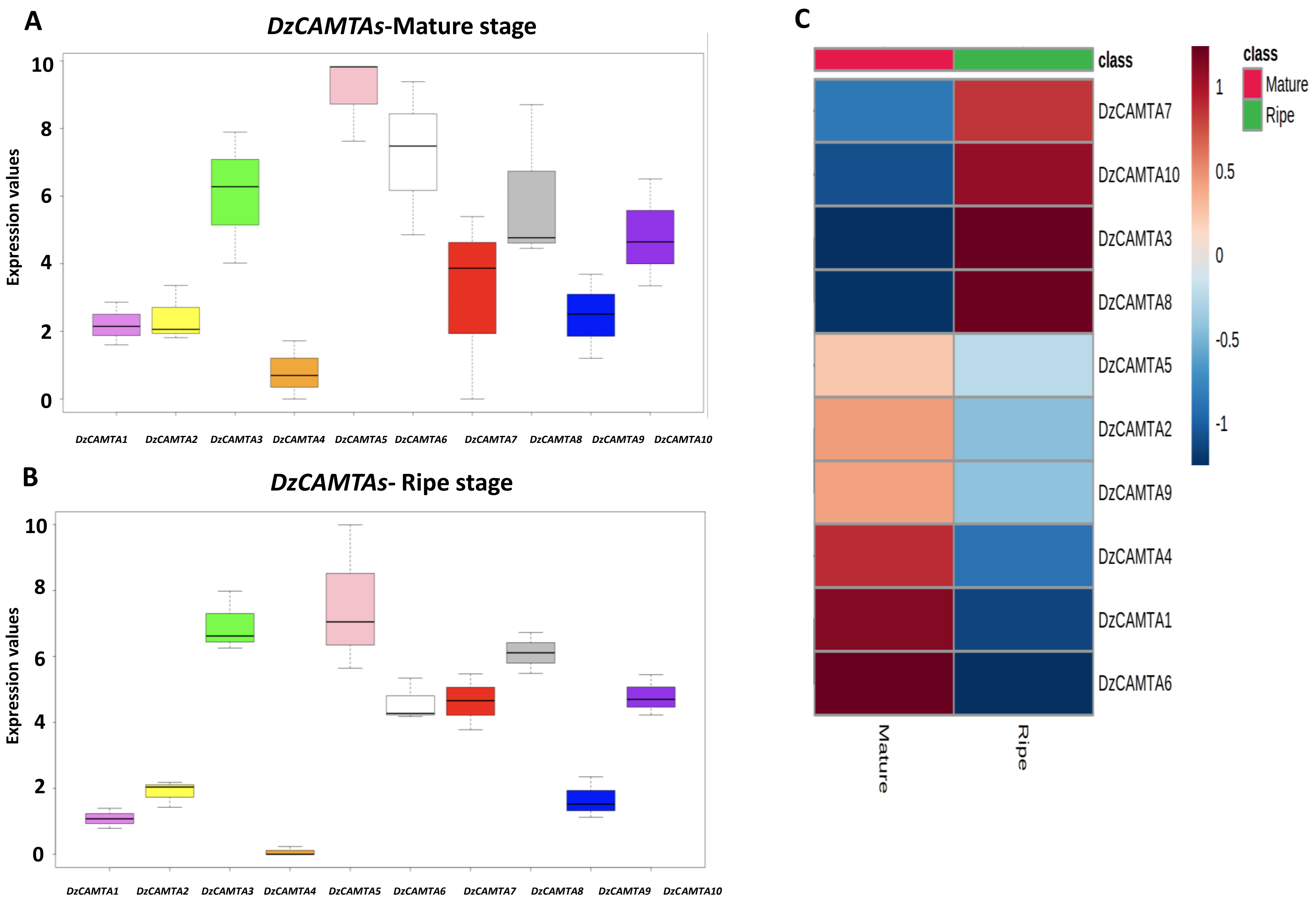

Additional file 5: Expression profile of *DzCAMTA*s during post-harvest ripening. Variation in expression of 10 *DzCAMTA*s at **(A)** mature stage and **(B)** ripe stage of *Durio Zibethinus* was visualized by box plot. Each *DzCAMTA* is represented with a different color. The central line for each box plot indicates the median value. The expression values correspond to the RPKM. **(C)** Expression profiles of 10 *DzCAMTA*s in mature and ripe stages of *Durio Zibethinus* pulp. Data was sum normalized, log transformed, and auto scaled.
