# Supplementary material for: Comprehensive genome-wide analysis of calmodulin-binding transcription activator (CAMTA) in Durio zibethinus and identification of fruit ripening-associated DzCAMTAs
Source: BMC Genomics. 2021 Oct 14;22:743. doi: 10.1186/s12864-021-08022-1 (PMC8518175; doi:10.1186/s12864-021-08022-1)
Supplement: Supplementary file 6 — Additional file 6. Functional and GO annotation of DzCAMTA3 and DzCAMTA8 interacting genes. MapMan based functional classification of DzCAMTA3PinG, DzCAMTA3NinG, DzCAMTA8PinG, and DzCAMTA8NinG. (A) RNA biosynthesis transcriptional regulation. (B) Multi-process regulation. (C) Coenzyme metabolism, secondary metabolism and redox homeostasis. (D) Solute transport. (E) Phytohormone action. (F) Protein modification. (G) Carbohydrate metabolism. (H)Cell wall organization. (I) External stimuli response. (J) Nutrient uptake. (K) Photosynthesis. The scale represents expression values in log2. [file 12864_2021_8022_MOESM6_ESM.pdf]

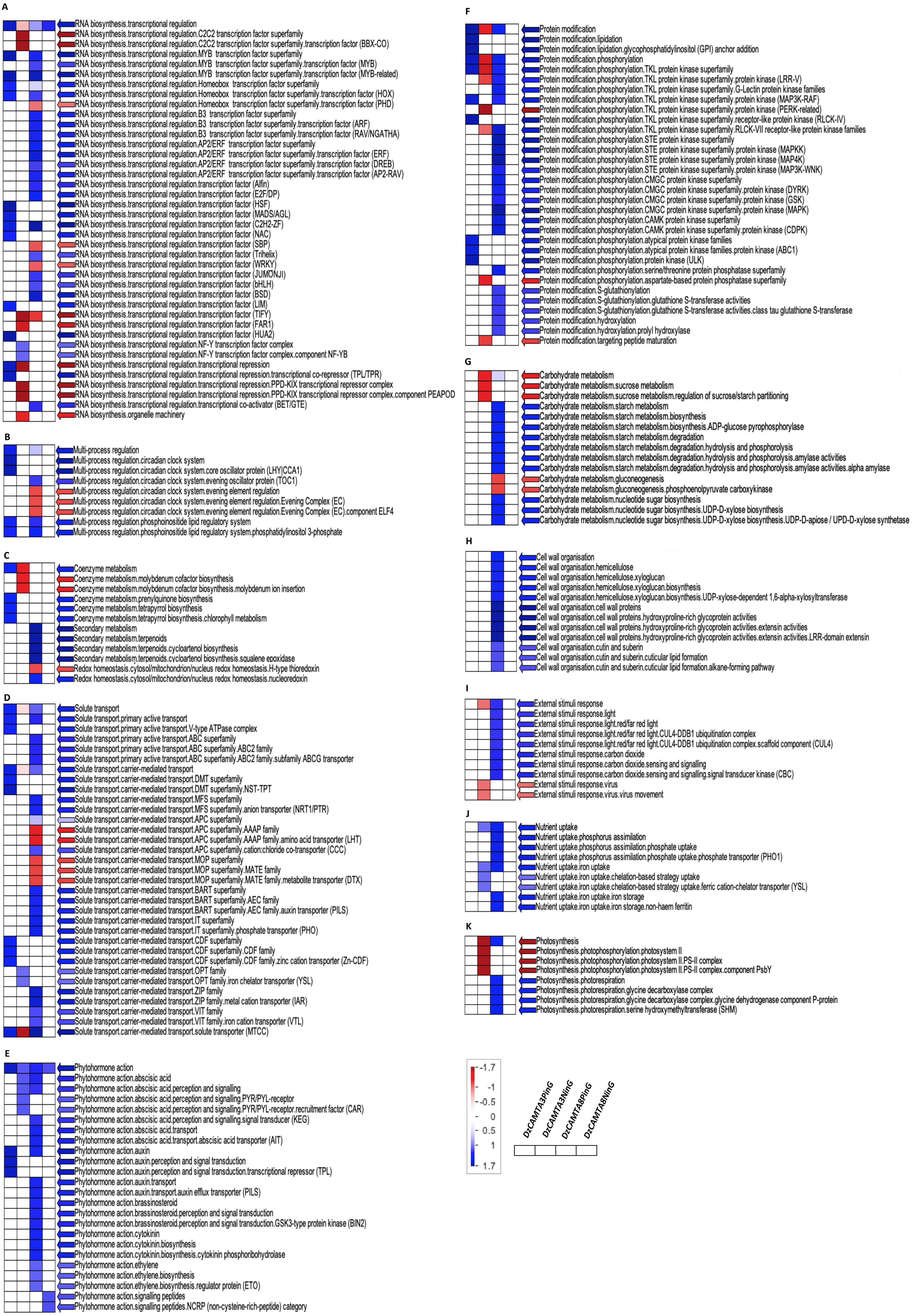

Additional file 6: Functional and GO annotation of *DzCAMTA3* and *DzCAMTA8* interacting genes. MapMan based functional classification of *DzCAMTA3PinG*, *DzCAMTA3NinG*, *DzCAMTA8PinG*, and *DzCAMTA8NinG*. **(A)** RNA biosynthesis transcriptional regulation. **(B)** Multi-process regulation. **(C)** Coenzyme metabolism, secondary metabolism and redox homeostasis. **(D)** Solute transport. **(E)** Phytohormone action. **(F)** Protein modification. **(G)** Carbohydrate metabolism. **(H)** Cell wall organization. **(I)** External stimuli response. **(J)** Nutrient uptake. **(K)** Photosynthesis. The scale represents expression values in log_2.
