# Supplementary material for: Comprehensive genome-wide analysis of calmodulin-binding transcription activator (CAMTA) in Durio zibethinus and identification of fruit ripening-associated DzCAMTAs
Source: BMC Genomics. 2021 Oct 14;22:743. doi: 10.1186/s12864-021-08022-1 (PMC8518175; doi:10.1186/s12864-021-08022-1)
Supplement: Supplementary file 8 — Additional file 8. GO annotation of DzCAMTA8PinG, and DzCAMTA8NinG with respect to biological processes, molecular functions and cellular components, respectively. [file 12864_2021_8022_MOESM8_ESM.pdf]

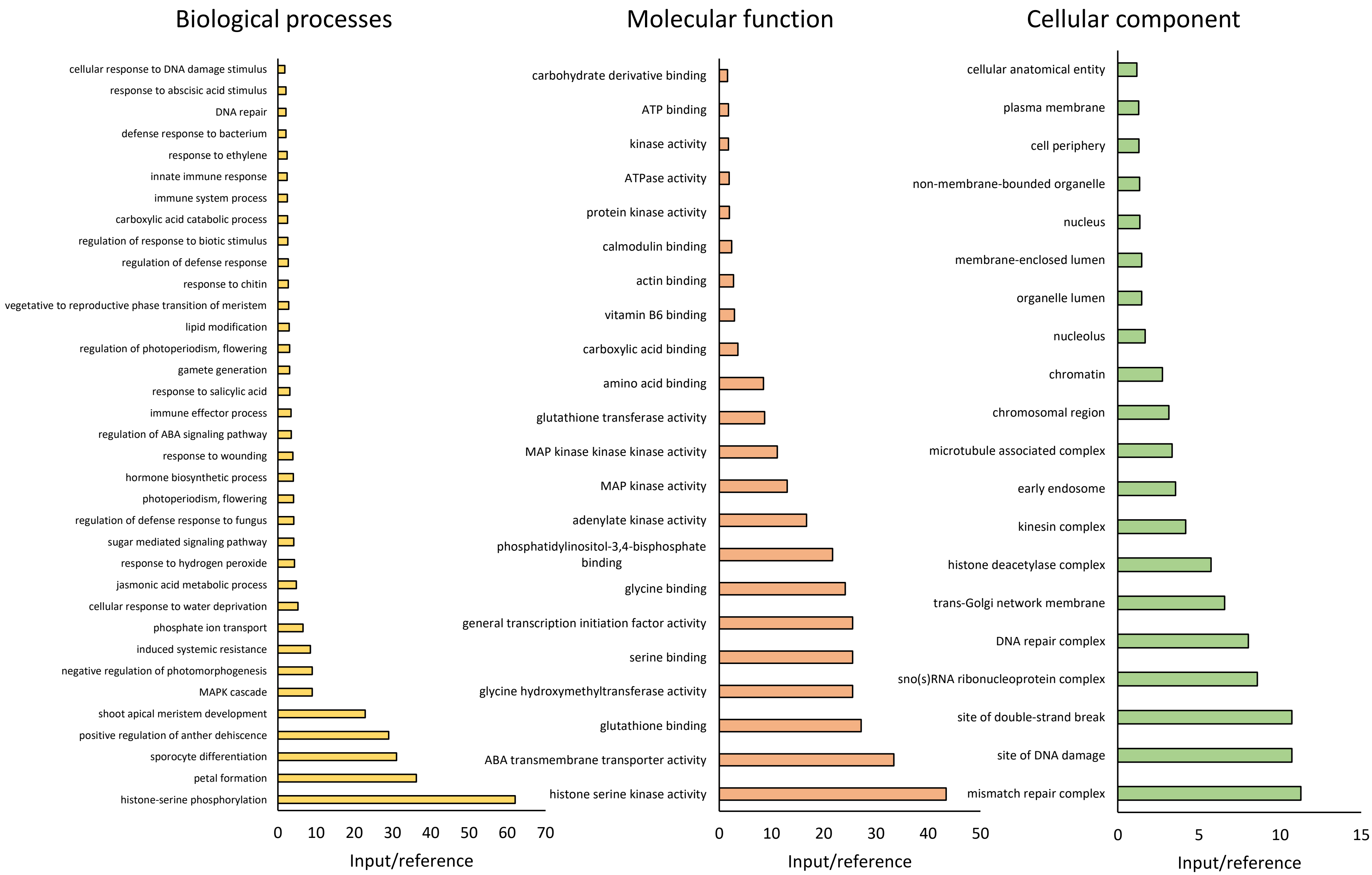

Additional file 8: GO annotation of *DzCAMTA8PinG*, and *DzCAMTA8NinG* with respect to biological processes, molecular functions and cellular components, respectively.
