# Supplementary material for: Comprehensive genome-wide analysis of calmodulin-binding transcription activator (CAMTA) in Durio zibethinus and identification of fruit ripening-associated DzCAMTAs
Source: BMC Genomics. 2021 Oct 14;22:743. doi: 10.1186/s12864-021-08022-1 (PMC8518175; doi:10.1186/s12864-021-08022-1)
Supplement: Supplementary file 9 — Additional file 9. GO functional annotation for the 10 DzCAMTAs. The different colors represent the proportions of various GO terms. [file 12864_2021_8022_MOESM9_ESM.pdf]

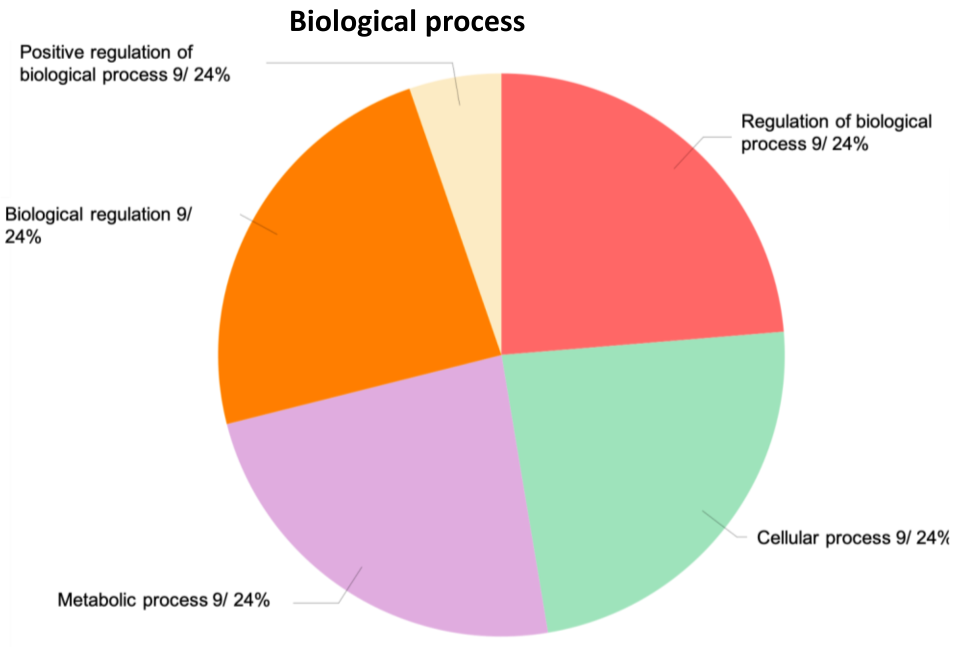

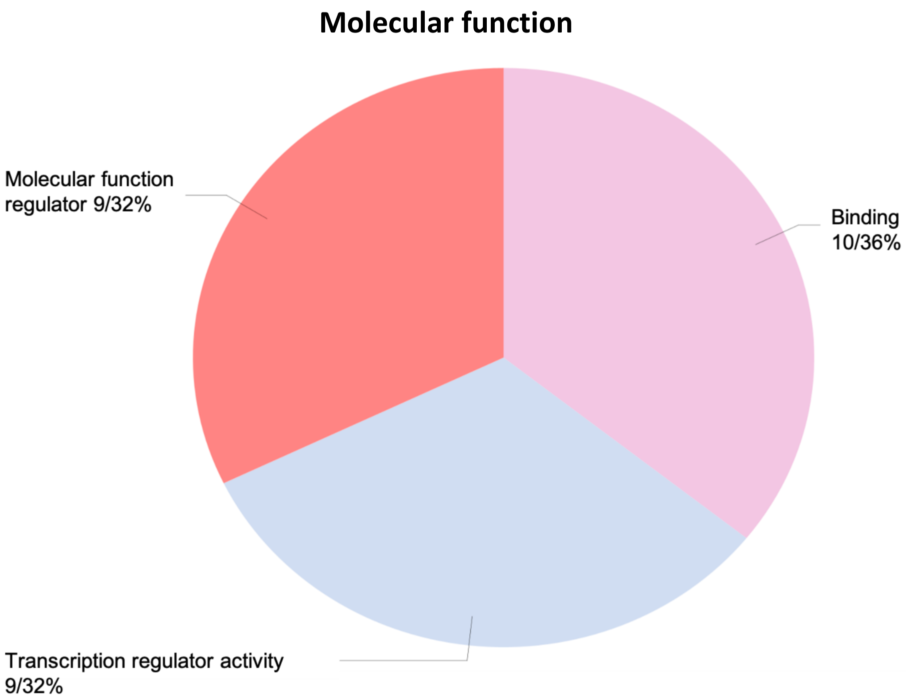

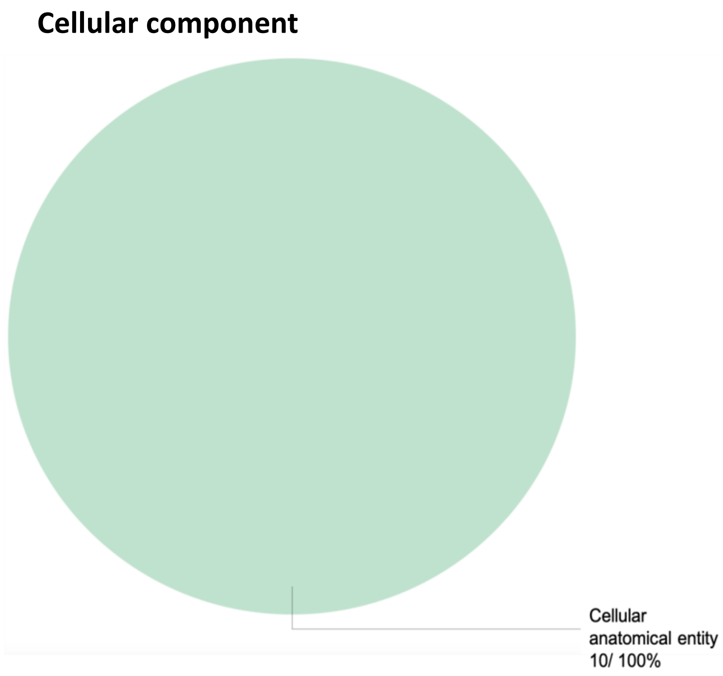

Additional file 9: GO functional annotation for the 10 *DzCAMTAs*. The different colors represent the proportions of various GO terms.
