# Supplementary material for: Comprehensive genome-wide analysis of calmodulin-binding transcription activator (CAMTA) in Durio zibethinus and identification of fruit ripening-associated DzCAMTAs
Source: BMC Genomics. 2021 Oct 14;22:743. doi: 10.1186/s12864-021-08022-1 (PMC8518175; doi:10.1186/s12864-021-08022-1)
Supplement: Supplementary file 11 — Additional file 11. Schematic representation of the role of ripening associated DzCAMTA3, and DzCAMTA8 in the regulatory network of durian fruit ripening. [file 12864_2021_8022_MOESM11_ESM.pdf]

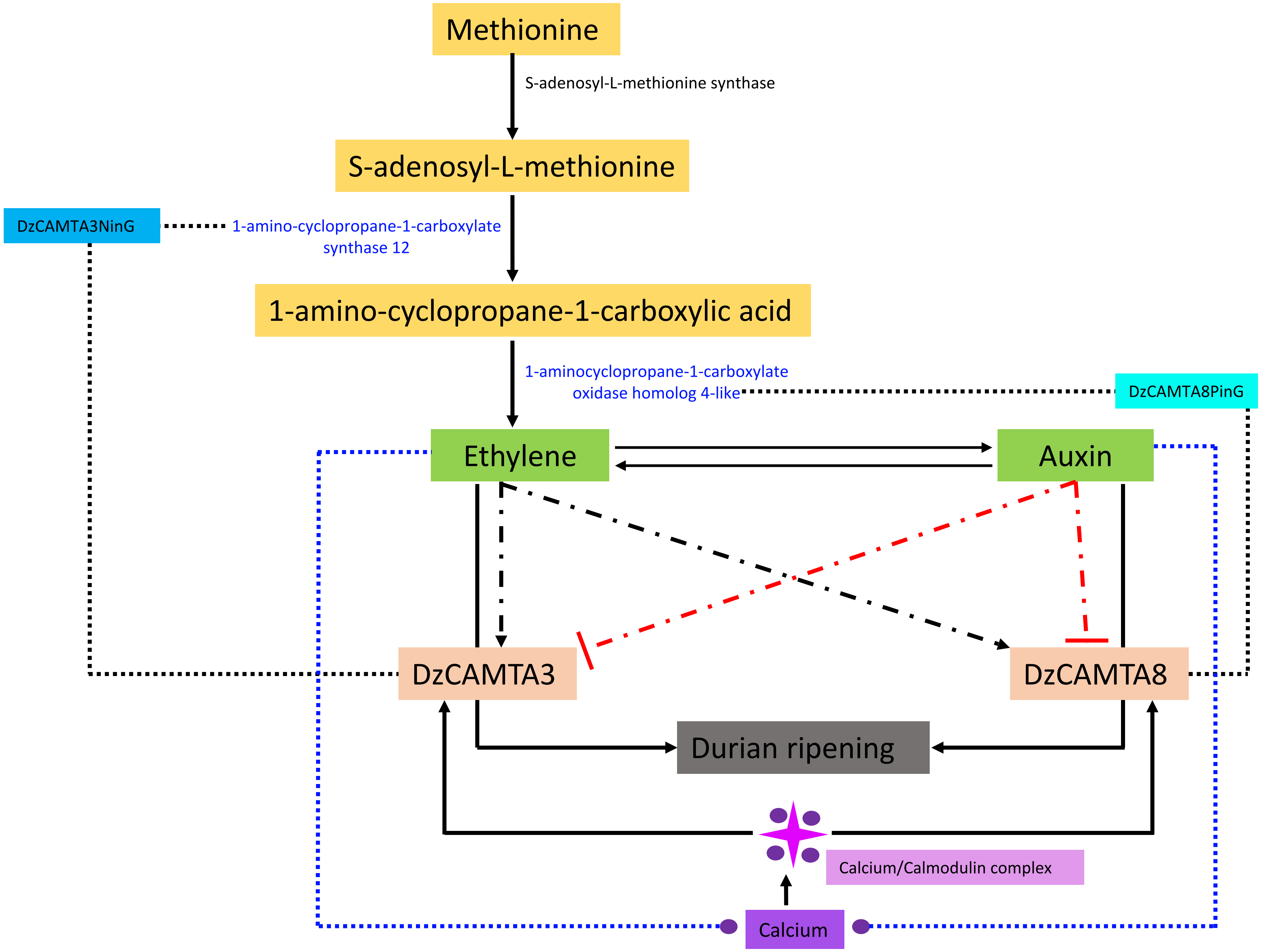

Additional file 11: Schematic representation of the role of ripening associated *DzCAMTA3*, and *DzCAMTA8* in the regulatory network of durian fruit ripening.
